# Protein Profiling in Serum and Cerebrospinal Fluid Following Complex Surgery on the Thoracic Aorta Identifies Biological Markers of Neurologic Injury

**DOI:** 10.1007/s12265-018-9835-8

**Published:** 2018-10-26

**Authors:** Rickard P. F. Lindblom, Qiujin Shen, Sofie Axén, Ulf Landegren, Masood Kamali-Moghaddam, Stefan Thelin

**Affiliations:** 1 Department of Cardiothoracic Surgery and Anesthesia, Uppsala University Hospital, SE-751 85 Uppsala, Sweden; 2 Department of Surgical Sciences, Section of Thoracic Surgery, Uppsala University, Uppsala, Sweden; 3 Department of Immunology, Genetics and Pathology, Science for Life Laboratory, Uppsala University, Uppsala, Sweden

**Keywords:** Thoracic aortic disease, Cardiovascular surgery, Neurologic injury, Biomarkers

## Abstract

Surgery on the arch or descending aorta is associated with significant risk of neurological complications. As a consequence of intubation and sedation, early neurologic injury may remain unnoticed. Biomarkers to aid in the initial diagnostics could prove of great value as immediate intervention is critical. Twenty-three patients operated in the thoracic aorta with significant risk of perioperative neurological injury were included. Cerebrospinal fluid (CSF) and serum were obtained preoperatively and in the first and second postoperative days and assessed with a panel of 92 neurological-related proteins. Three patients suffered spinal cord injury (SCI), eight delirium, and nine hallucinations. There were markers in both serum and CSF that differed between the affected and non-affected patients (SCI; IL6, GFAP, CSPG4, delirium; TR4, EZH2, hallucinations; NF1). The study identifies markers in serum and CSF that reflect the occurrence of neurologic insults following aortic surgery, which may aid in the care of these patients.

**Electronic supplementary material:**

The online version of this article (10.1007/s12265-018-9835-8) contains supplementary material, which is available to authorized users.

## Introduction

Surgery of the thoracic aorta is life-saving, although not without risks. Besides the surgical risk of major bleeding, devastating central nervous system (CNS) complications in the form of stroke or paraplegia are not uncommon.

There has been a rapid development of endovascular surgical approaches of correcting thoracic aortic disease during the recent decade [7]; however, in situations of complex pathology that affects the arch vessels, open surgery with hybrid approaches are perhaps the currently most preferred [28].

Apart from the risk of disturbing the cerebral circulation during reconstruction of the arch vessels, there is also a risk of disrupting the spinal blood flow during surgery of the arch and descending aorta. There exist a number of adjunct therapies that can be undertaken with the aim to prevent spinal injury, including elevated perfusion pressures, moderate/deep hypothermia, placement of intrathecal drainage, perioperative neurophysiologic monitoring, and to surgically avoid compromising more than one vascular territory that supplies the spinal cord (subclavian-intercostal-lumbar) [13, 17, 23, 34].

Early detection of CNS symptoms with subsequent intervention can potentially prevent injury from becoming manifest, for instance by elevating perfusion pressure or lowering intracranial/spinal pressure (ICP). It is therefore of essence to detect changes immediately [13]. However, these patients are often circulatory unstable in the initial phase after surgery, which makes it unsuitable to move them for radiological imaging. Furthermore, radiological exams early after ischemic injury may be unreliable [11]. The patients are most often intubated and thereby sedated for many hours after surgery, sometimes even days, which makes clinical examinations challenging. These factors taken together make it difficult to detect, let alone intervene against ischemic injury.

For many acute [4] and chronic [3, 32] neurological diseases, establishment of biomarkers has allowed improvement of diagnose and prediction of disease course, but biological markers are under-developed for patients undergoing aortic surgery. In the current study, we studied serum and cerebrospinal protein profiles in patients operated on the thoracic aorta, where a significant risk of developing neurologic injury was present. The protein analysis was directed against neuronal-related proteins with the aim to identify new markers of value in the perioperative care of this group of patients.

## Methods

### Patients

All patients with pathology of the thoracic aorta operated at the Department of Cardiothoracic Surgery and Anesthesia at Uppsala University Hospital, with substantial risk for developing SCI, and who consented to having a spinal drain were after informed written consent included in the study. All patients consented to having the intrathecal catheter placed, in the evening before surgery, but in three patients, this was not successful. The drainage was set to automatically drain if ICP was over 10-cm H_2_O.

### Surgery

Four patients were operated with posterolateral thoracotomy, whereof one patient was closed without further procedure, i.e., only exploratory. The three patients that were operated were so using standard woven grafts with partial bypass, with femoral cannulation and extra-corporeal perfusion of the lower body, at 34 or 35 °C.

One patient was operated with a supracoronary woven graft, via median sternotomy and hemi-arch reconstruction using retrograde cerebral perfusion and moderate to deep hypothermia (21 °C). Sixteen patients were operated with Frozen Elephant Trunc (FET) grafts; 7 E-vita (Jotec, Hechingen, Germany) and nine thoraflex hybrid (Vascutec, Terumo, Renfrewshire, Scotland). These were all operated with a median sternotomy and selective antegrade cerebral perfusion with circulatory arrest and moderate hypothermia (20–25 °C). Nine out of the 16 FET patients (56.3%) were preoperatively deviated with the left subclavian artery transposed to the left carotid artery. The innominate and left carotid artery were re-implanted in all cases perioperatively into the FET graft. In the seven none-deviated subclavian arteries, the left subclavian artery was reimplanted in the FET graft in 2 (12.5%) cases, whereas it was ligated perioperatively in five patients (31.3%). This means that 11 of 16 FET patients (68.8%) had the left subclavian reconnected.

Two patients were operated with TEVAR, with covered stent grafts in the descending aorta (Gore Tag, 34 × 100 mm in one patient and two grafts 31 × 150 and 37 × 200 mm in the other patient). Both were deemed at risk of SCI as one had a long section of the aorta stented, and the other patient had several stent grafts earlier in the descending aorta with leakage in-between the grafts. These patients are included in the clinical outcomes, but not in the postoperative protein analysis, see below.

### Sample Preparation

Both serum and cerebrospinal fluid (CSF) samples were obtained preoperatively and at 8 am in the first and second postoperative mornings. See Supplementary data for details.

### Clinical Data

The clinical data of the patients is summarized in Tables 1, 2 and 3 and described in the Supplementary data.

**Table 1:** Preoperative clinical data of the patients

Patients (*n* = 23)		
Mean age (SD), year	59.8	(11.6)
	*n*	%
Sex		
Male	18	78.3
Female	5	21.7
Hypertension		
Yes	22	95.7
No	1	4.3
Diabetes		
Yes	4	17.4
No	19	82.6
COPD		
Yes	5	21.7
No	18	78.3
EF		
> 45	19	82.6
30–45	4	17.4
< 30	0	0
Class of heart failure		
NYHA I	3	13.0
NYHA II	9	39.1
NYHA IIIa	5	21.7
NYHA IIIb	1	4.3
N/A	5	21.7
Previous cardiac surgery		
Yes	10	43.5
No	13	56.5
Absolute GFR (Lund-Malmö)		
Normal (GFR > 85)	8	34.8
Moderate (50–85)	12	52.2
Severe (< 50)	3	13.0

COPD: chronic obstructive pulmonary disease, EF: ejection fraction, NYHA: New York Heart Association, GFR: glomerular filtration rate

**Table 2:** Perioperative clinical data of the patients

	*n*	%
Diagnose		
Acute type-A aortic dissection	2	8.7
Chronic type-A aortic dissection	8	34.8
Chronic type-B aortic dissection	4	17.4
Aortic aneurysm	9	39.1
Type of procedure		
FET	16	69.6
TEVAR	2	8.7
Woven graft ascending aorta	1	4.3
Woven graft descending aorta	3	13.0
Exploratory Thoracotomy	1	4.3
Preoperative subclavia deviation		
Yes	9	39.1
No	14	60.9
Spinal drain	20	87.0
Heart valve surgery	5	21.7
CABG	0	0
Dimension of aorta		
Mean (SD), mm	62.8	(10.7)
ECC-time (minutes)		
Mean duration (*n* = 20)	293.4	
Mean aortic cross-clamp (*n* = 17)	148.7	

CABG, coronary artery by-pass grafting; ECC, extra-corporeal circulation; FET, frozen elephant trunk; TEVAR, thoracic endovascular aortic repair

**Table 3:** Postoperative clinical data of the patients

	*n*	%
30-Day mortality	0	0
Deceased (within follow-up)	2	8.6
Neurological sequelae		
Total	14	60.9
Spinal cord injury	3	13.0
Delirium	8	34.8
Hallucination	9	39.1
Reoperation		
Total	7	30.4
Bleeding	5	21.7
Cardiac tamponade	2	8.7
Infection	0	0
Dialysis	2	8.7
Atrial fibrillation	12	52.2
Pleural drainage	3	13.0
Pericardiocentesis	1	4.3
Stroke	0	0
Postlumbar puncture headache	4	18.2

### Protein Measurements

The levels of 92 proteins with relevance to neurology were measured simultaneously by multiplex proximity extension assay (PEA). The assay was performed according to the manufacturer’s recommendations (www.olink.com). See Supplementary data for details.

### Statistics

Among the serum and CSF samples from 23 patients, postoperative samples from three patients were removed for analysis because two had endovascular treatment and one patient only underwent explorative thoracotomy. These were excluded as the use of extracorporeal circulation and the much more extensive surgery in remaining patients had such a profound biochemical impact, which made comparisons unjustifiable. One postoperative serum sample was excluded, since the levels of all the proteins in this sample were highly aberrant from the other samples, most likely due to technical reasons at sampling. Among the 92 proteins included in the panel, five were excluded due to suspected antibody cross-activity. Another 21 proteins were excluded because the detectable percentages of these proteins were less than 50% in both serum and CSF samples in all the pre- and postoperative groups, including CX3CR1, DCLK1, HES1, HOXB1, JAG1, KLF4, MAPT, MSI2, NES, NGFR, NOG, OLIG2, PTCH1, RTN4RL1, S100B, SLC1A3, SOX1, SOX5, SOX11, SOX21, and TPH1 (Table S1). An individual assessment of these 21 proteins, verified that in 20 of 21 cases, the levels were low in all samples. However, CX3CR1 differed and the levels were low in general, but were to a higher degree detectable in the patients with postoperative SCI (Fig. S2). In summary, 66 proteins in 59 serum and 50 CSF samples were included in the finals analysis.

In general, the groups were small, especially those with neurological complications. Therefore, for the analysis of protein levels in the patients with neurological complications, postoperative days 1 and 2 were clustered together in the patients that either developed neurological complications and those that did not.

Differences in pre- and postoperative protein levels were tested by non-parametric Mann-Whitney *U* test. False discovery rate (FDR) was applied to adjust for multiple testing. A *p* value smaller than 0.05 (two-tailed) was considered as significant. Data analysis and interpretation were performed in R software (www.r-project.org). The exact *p* values are for clarity shown in the tables and not in the individual graphs.

To verify the consistency and assess potential intraassay variability, several samples were run in duplicates and the correlation coefficient analyzed with Pearson’s test. The *r* values were between 0.996 and 0.998, indicating a high reproducibility of the assay (data not shown).

### Study Approval

The study was approved by the local ethical committee (Regionala Etikprövningsnämnden i Uppsala) under permit number 2012/296.

## Results

### Patient Characteristics Preoperatively

Twenty-three patients were included during a period of 39 months. Mean follow-up was 38 months (range 18 to 58). The mean age was 59.8 years (± 11.6 SD), although the distribution spread over a range from 26 to 77 years. The included diagnoses were two acute type-A dissections, eight chronic type-A dissections, four chronic type-B dissections, and nine aneurysms. The mean maximum dimension of the aorta was 63 ± 11 mm.

Two patients were operated with TEVAR of the descending aorta, 16 patients with frozen elephant trunk (FET), four patients with standard woven vascular grafts (one ascending/three descending), and one patient was only operated with an exploratory thoracotomy, and then closed due to porcelain aorta. In three patients (13%), it was not possible to place a spinal drain; nine patients, all candidates for FET (39% of all patients, 56% of the FET), were preoperatively deviated from the left carotid to subclavian artery.

All patients but one had hypertension (96%), 12 (52%) were or had been smokers, and four (17%) had reduced left ventricular function (LVEF 30–45%). Ten (44%) had previous cardiac surgery with opened pericardium. Twelve patients (52%) had moderately reduced renal function (GFR 50–85 ml/min) and three (13%) had markedly reduced renal function (GFR < 50 ml/min). See Tables 1 and 2 for details.

### Postoperative Data

#### General Parameters

There was no 30-day mortality; however, two patients died during follow-up, both FET operated, one 7 months postoperatively, and one 17 months postoperatively. The causes of death were aortic rupture distal to the thoraflex graft and multi-organ failures after graft infection in the thoracoabdominal endovascular stent grafts used in the extension of the FET graft, respectively. The mean ECC (extracorporeal circulation) time was 293.4 min, and the mean aortic cross-clamp time was 148.7 min. The mean amount of blood products used intraoperatively was 1990-ml packed red blood cells, 1780-ml fresh frozen plasma, and 950-ml platelet concentrate. Seven patients (30%) were re-operated for bleeding or tamponade and one (4.3%) required pericardiocentesis; no patients were re-operated for infection. Two patients (8.7%) required postoperative dialysis. Atrial fibrillation was common, 12 patients (52%). See Table 3 for details.

#### Neurological Complications

Three patients suffered ischemic spinal cord injury (SCI; 13%), although one patient was paraparetic before start of operation (acute type-A dissection), so only two were related to the surgery (8.7%), one open descending operation, and one FET (who did not have a deviated subclavia). Eight patients (35%) developed delirium, nine patients (39%) had hallucinations, and four (18%) developed spinal headache. No patients developed stroke. A few patients had more than one neurological complication. In summary, 14 patients (60.9%) suffered from any type of the above-mentioned neurological complication postoperatively. See Table 3 for details.

### Biochemical Data

As expected, there was an obvious difference of protein profiles between serum and CSF, as shown in principal component analysis and heatmap (Fig. S1a and b). Levels of 50 out of 66 proteins were significantly different after *p* values adjusted with 5% FDR or 40 after Bonferroni correction for multiple testing (*p* < 0.05/60) (Table S2a and b).

**Fig. 1:**
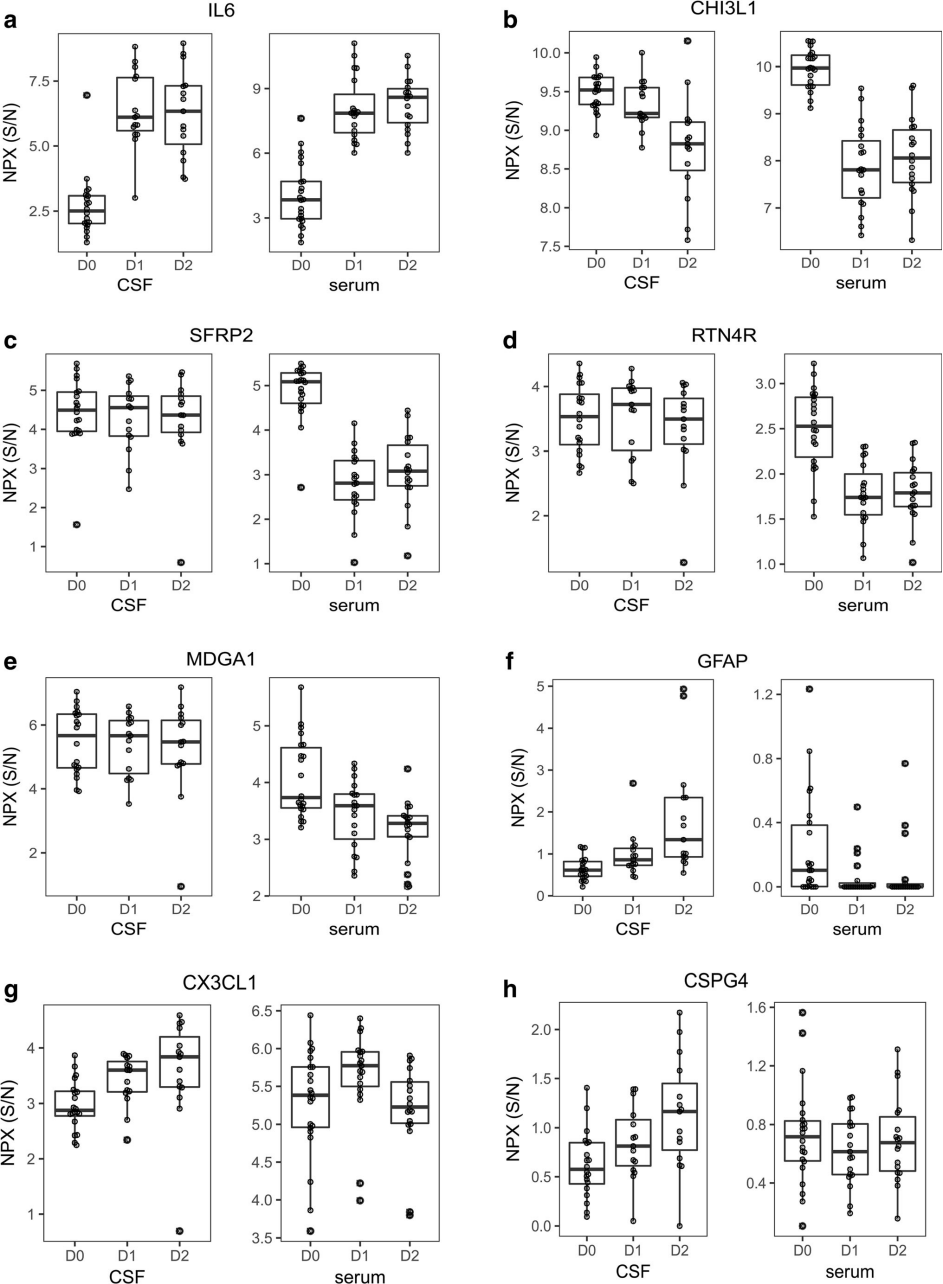
The proteins differing most significantly in serum (**a**–**e**) and CSF (**a**, **b**, **f**–**h**) postoperatively compared with preoperatively. D0 = preoperatively, D1 = postoperative day 1, D2 = postoperative day 2. NPX S/N: normalized expression as explained in methods. N = serum; D0:22, D1:19, D2:18, CSF; D0:20, D1:15, D2:15

There were also large differences between the preoperative and postoperative protein profiles in the patient population as a whole (Fig. 1). As the focus of the current study was on the patient group with neurological complications, no further description of the general patient population is given here, but can be found in the Supplementary data.

#### Patients with Postoperative Spinal Cord Injuries

**Fig. 2:**
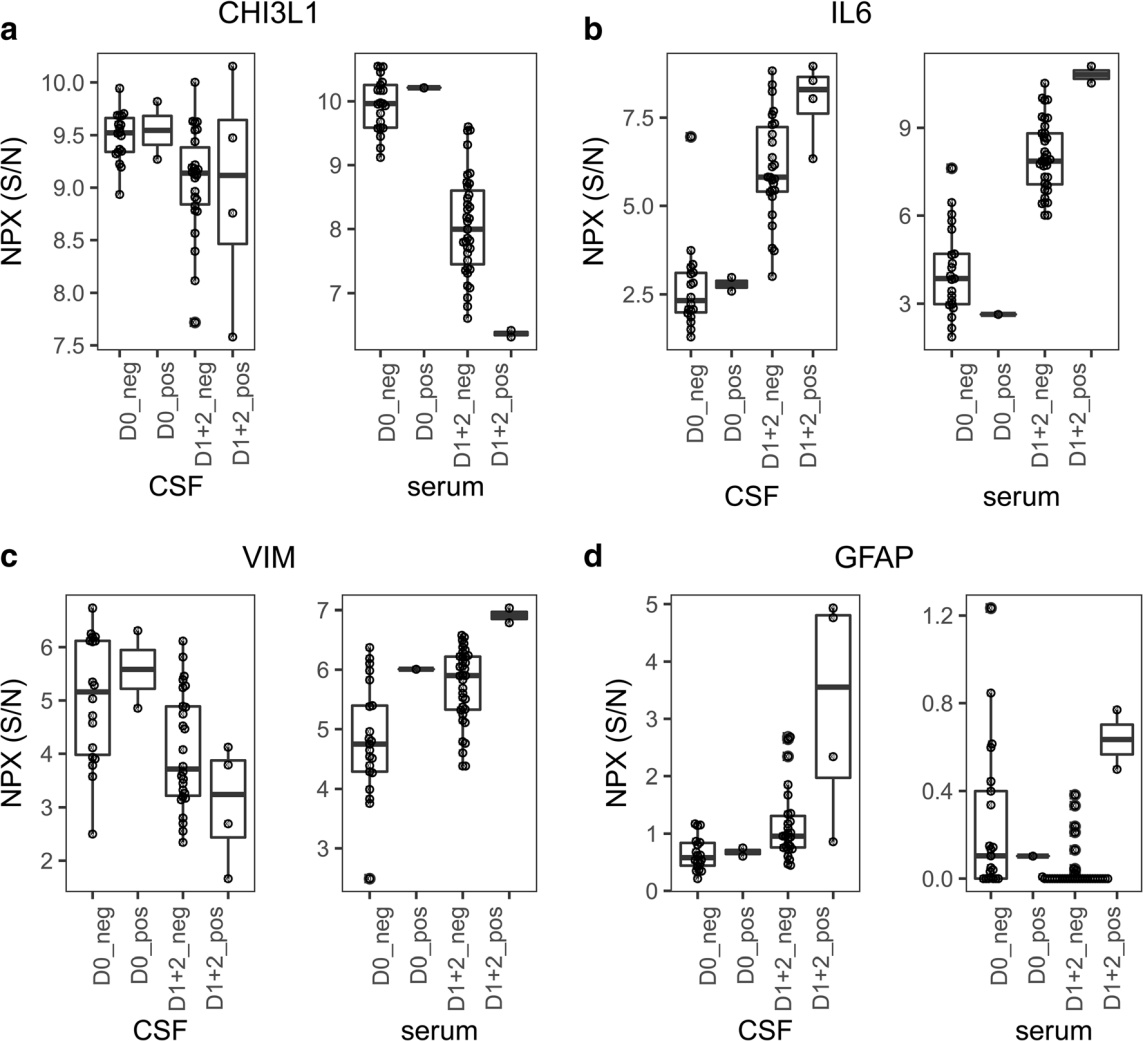
The proteins differing most significantly in serum (**a**–**e**) and CSF (**e**–**j**) in the patients that suffered postoperative spinal cord injury (SCI) compared to those that did not postoperatively. NPX S/N: normalized expression as explained in methods. D0_neg = preoperative levels in patients with no postoperative injury, D0_pos = preoperative levels in patients postoperatively injured, D1 + D2_neg = all patients that did not suffer SCI postoperatively. D1 + D2_pos = all patients that suffered SCI postoperatively. N = CSF: D0_neg:18, D0_pos:2, D1 + D2_neg:26, D1 + D2_pos:4, Serum n = D0_neg:21, D0_pos:1, D1 + D2_neg:35, D1 + D2_pos:2

There were few samples from patients with SCI, but the levels of 15 proteins differed between the injured and non-injured patients in serum at unadjusted *p* < 0.05 and 4 at *p* < 0.01; CHI3L1, VIM, IL6, and GFAP (Fig. 2a–d, Table 4). However, no protein was significant between the injured and non-injured patients after FDR correction (Table 4).

**Table 4:** Spinal cord injuries, postoperative levels non-injured compared with injured, serum

Serum protein	mean ± SD_D1 + D2_neg	mean ± SD_D1 + D2_pos	mean_D1 + D2 pos_D1 + D2 neg	P_D1 + D2 neg vs. D1 + D2 pos	FDR_adj_ P_D1 + D2 neg vs. D1 + D2 pos
CHI3L1	8.06 ± 0.8	6.37 ± 0.1	− 1.69505	0.0030	0.0661
IL6	8.01 ± 1.2	10.81 ± 0.4	2.797483	0.0030	0.0661
VIM	5.73 ± 0.6	6.91 ± 0.2	1.178842	0.0030	0.0661
GFAP	0.04 ± 0.1	0.63 ± 0.2	0.593404	0.0041	0.0684
SIRT2	4.25 ± 0.9	6.51 ± 1	2.258325	0.0120	0.1014
CDCP1	3.95 ± 0.6	5.14 ± 0.1	1.191632	0.0120	0.1014
MMP2	6.13 ± 0.2	6.53 ± 0.1	0.396763	0.0120	0.1014
GAD2	0.09 ± 0.2	0.52 ± 0	0.43172	0.0123	0.1014
GMFB	0.1 ± 0.2	0.84 ± 0.1	0.742681	0.0148	0.1083
PMP2	1.69 ± 1.1	4.52 ± 0.7	2.826157	0.0244	0.1372
MGMT	4.71 ± 1.2	6.73 ± 0.6	2.025262	0.0270	0.1372
ITGA2	2.33 ± 0.3	2.94 ± 0.4	0.610513	0.0270	0.1372
CSPG4	0.64 ± 0.3	1.07 ± 0.1	0.429215	0.0270	0.1372
DLL1	10.9 ± 0.4	11.34 ± 0	0.43828	0.0480	0.2114
PXN	3.46 ± 0.7	4.42 ± 0.1	0.962561	0.0480	0.2114

D0: preoperatively; D1 + D2: postoperative day 1 and postoperative day 2 combined; neg: no spinal cord injury; pos: spinal cord injury; FDR: false discovery rate

The levels of two proteins, IL6, and GFAP differed in CSF between patients with and without SCI at uncorrected *p* < 0.05, whereas none was significant after FDR correction (Fig. 2b, d, Table 5). IL6 was however clearly raised in the injured patients postoperatively compared to baseline also after *p* value correction (Fig. 2b, Table S3d). CX3CR1 showed an interesting pattern, with increased levels in the CSF of the injured compared to the non-injured postoperatively, but this did not quite reach statistical significance (Fig. S2).

**Table 5:** Spinal cord injuries, postoperative levels non-injured compared with injured, CSF

CSF protein	mean ± SD_D1 + D2_neg	mean ± SD_ D1 + D2_pos	mean_ D1 + D2 pos – D1 + D2 neg	P_D1 + D2 neg vs. D1 + D2 pos	FDR_adj_ P_D1 + D2 neg vs. D1 + D2 pos
IL6	6.07 ± 1.5	7.97 ± 1.1	1.90	0.0176	0.9072
GFAP	1.15 ± 0.6	3.23 ± 2	2.08	0.0444	0.9072

D0: preoperatively; D1 + D2: postoperative day 1 and postoperative day 2 combined; neg: no spinal cord injury; pos: spinal cord injury; FDR: false discovery rate

#### Patients with Postoperative Delirium

**Fig. 3:**
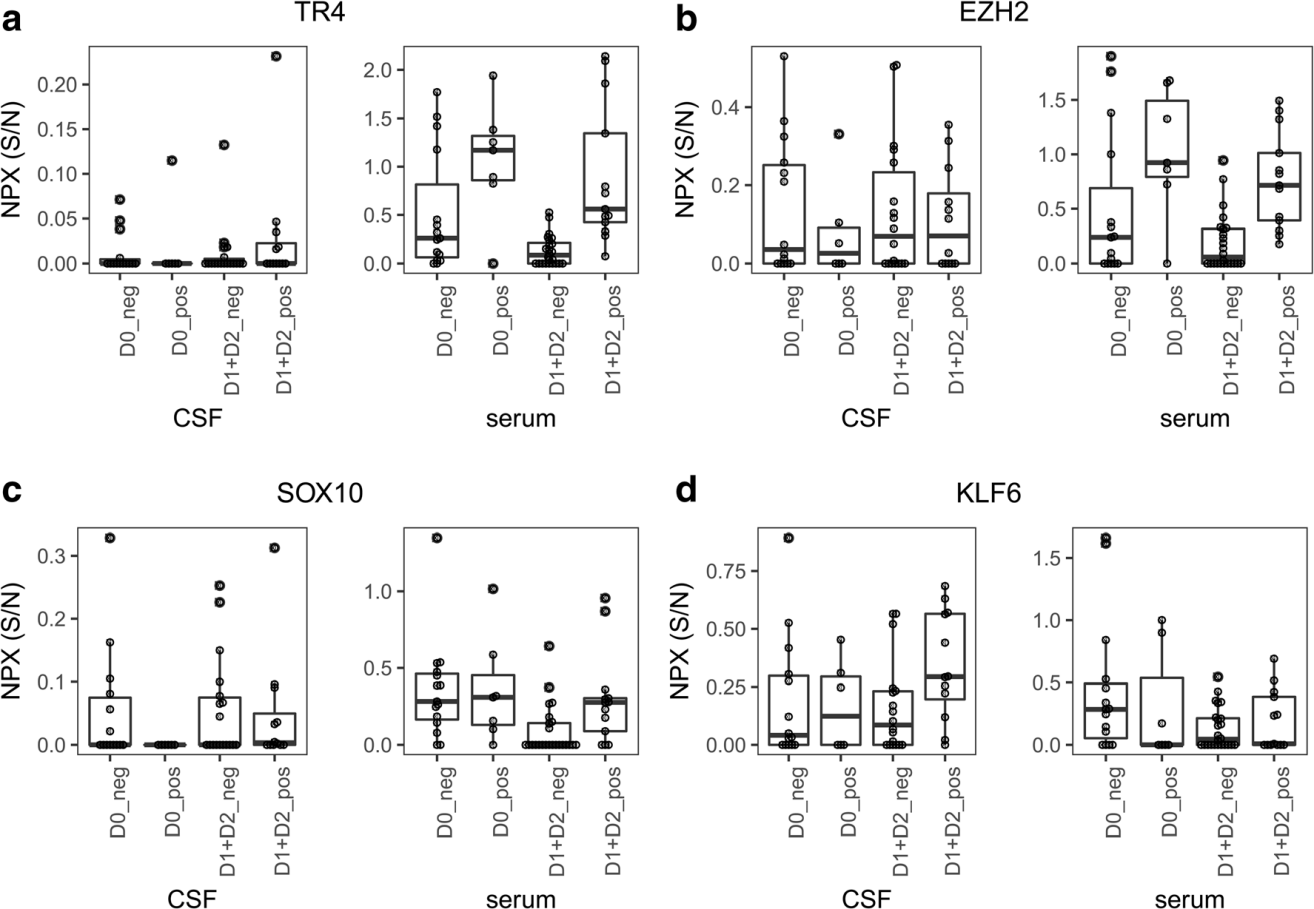
The proteins differing most significantly in serum (**a**–**c**) and CSF (**d**–**f**) in the patients that suffered postoperative delirium compared to those that did not postoperatively. NPX S/N: normalized expression as explained in methods. D0_neg = preoperative levels in patients with no postoperative delirium, D0_pos = preoperative levels in patients with postoperative delirium, D1 + D2_neg = all patients that did not suffer delirium postoperatively. D1 + D2_pos = all patients that suffered delirium postoperatively. N = CSF: D0_neg:14, D0_pos:6, D1 + D2_neg:18, D1 + D2_pos:12, Serum n = D0_neg:15, D0_pos:7, D1 + D2_neg:24, D1 + D2_pos:13

For delirium, the levels of three proteins differed in serum of affected compared to non-affected patients at uncorrected *p* < 0.01 (TR4, EZH2, and SOX10) and 15 at *p* < 0.05 (Fig. 3a–c, Table 6). TR4 and EZH2 were still significantly higher in patients with postoperative delirium compared to those without after FDR correction (Table 6). Interestingly, TR4 and EZH2 differed already preoperatively in the patients that developed delirium after operation (Fig. 3a–b). Five proteins (CHI3L1, IL6, SFRP2, PMP2, and RTN4R) differed in serum in patients with postoperative delirium compared to baseline with corrected *p* < 0.01 and 11 at *p* < 0.05 (Table S4c).

**Table 6:** Delirium, postoperative levels non-injured compared with injured, serum

Serum protein	mean ± SD_D1 + D2_neg	mean ± SD_D1 + D2_pos	mean_D1 + D2 pos_D1 + D2 neg	P_D1 + D2 neg vs. D1 + D2 pos	FDR_adj_ P_D1 + D2 neg vs. D1 + D2 pos
TR4	0.13 ± 0.2	0.89 ± 0.7	0.76	0.0000	0.0011
EZH2	0.19 ± 0.3	0.76 ± 0.4	0.57	0.0002	0.0050
SOX10	0.09 ± 0.2	0.3 ± 0.3	0.21	0.0076	0.1662
SMAD9	0.21 ± 0.2	0.49 ± 0.3	0.28	0.0105	0.1738
IL1B	0.16 ± 0.4	0.24 ± 0.3	0.08	0.0170	0.1890
IQGAP1	0.16 ± 0.2	0.55 ± 0.5	0.39	0.0212	0.1890
GMFB	0.09 ± 0.2	0.23 ± 0.2	0.14	0.0260	0.1890
CC2	0.17 ± 0.2	0.4 ± 0.3	0.23	0.0263	0.1890
BMI1	0.07 ± 0.1	0.24 ± 0.2	0.17	0.0298	0.1890
BMPR1B	0.12 ± 0.2	0.22 ± 0.2	0.10	0.0335	0.1890
SFRP2	2.8 ± 0.6	3.17 ± 1	0.37	0.0390	0.1890
SOX2	0.07 ± 0.1	0.26 ± 0.3	0.19	0.0395	0.1890
FABP7	0.02 ± 0.1	0.1 ± 0.1	0.09	0.0407	0.1890
GAD2	0.08 ± 0.2	0.19 ± 0.2	0.11	0.0428	0.1890
PTCh2	0.1 ± 0.1	0.18 ± 0.1	0.08	0.0430	0.1890

D0: preoperatively; D1 + D2: postoperative day 1 and postoperative day 2 combined; neg: no delirium; pos: delirium; FDR: false discovery rate

**Fig. 4:**
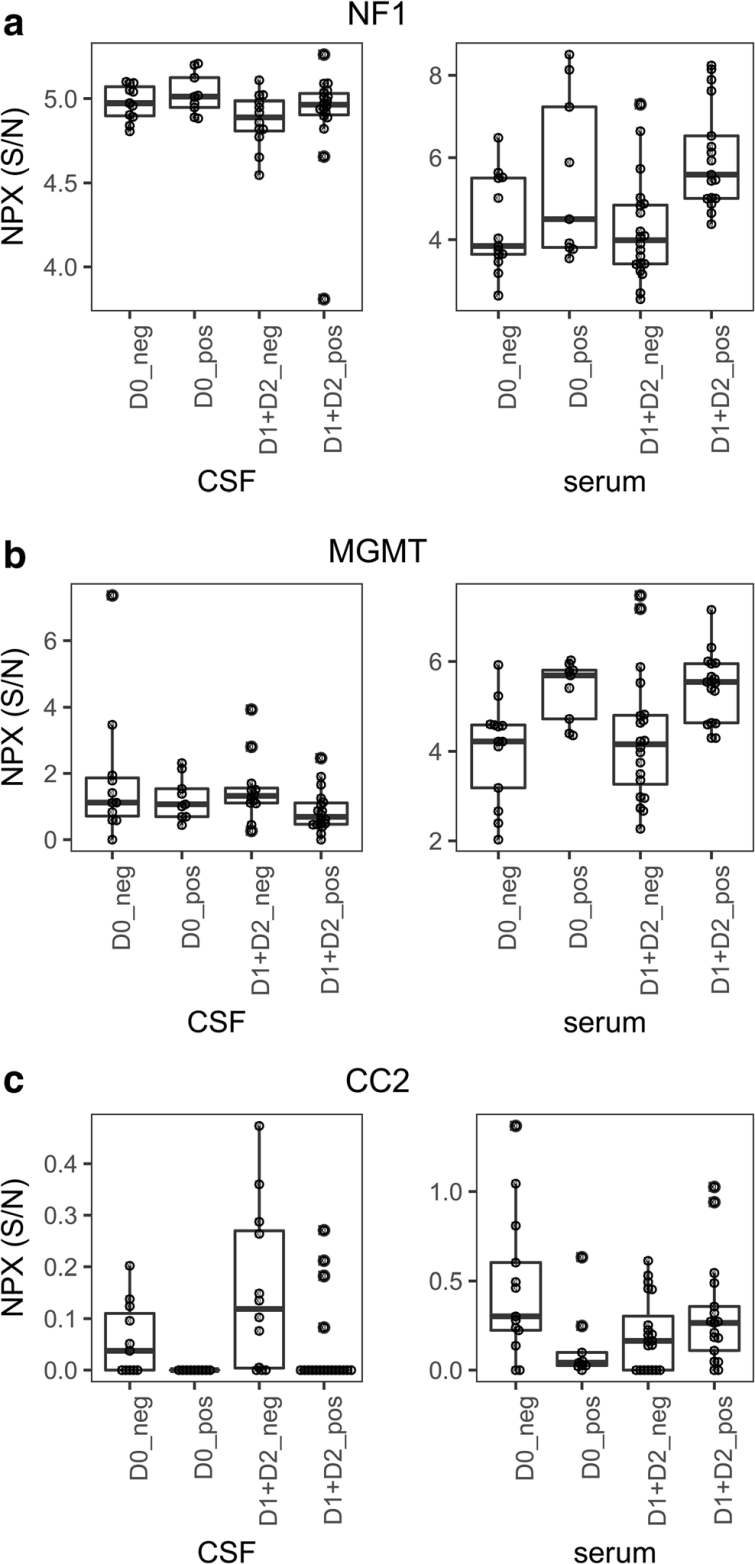
The proteins differing most significantly in serum (**a**–**b**) and CSF (**c**–**f**) in the patients that suffered postoperative hallucinations compared to those that did not postoperatively. NPX S/N: normalized expression as explained in methods. D0_neg = preoperative levels in patients with no postoperative hallucinations, D0_pos = preoperative levels in patients with in patients with postoperative hallucinations, D1 + D2_neg = all patients that did not suffer hallucinations postoperatively. D1 + D2_pos = all patients that suffered hallucinations postoperatively. N = CSF: D0_neg:11, D0_pos:9, D1 + D2_neg:12, D1 + D2_pos:18, Serum n = D0_neg:13, D0_pos:9, D1 + D2_neg:20, D1 + D2_pos:17

Only one protein, KLF6, differed between the postoperative CSF levels in patients with and without delirium at uncorrected *p* < 0.05 (Fig. 4d, Table 7). IL6, GFAP, CX3CL1, and ICAM1 were significantly increased compared to preoperatively in the patients with postoperative delirium (Table S4d), and CHI3L1 and VIM were significantly decreased postoperatively in the patients with delirium compared to preoperatively, but only IL6 significantly rose in the patients without delirium compared to preoperatively (Table S4b and d).

**Table 7:** Delirium, postoperative levels non-injured compared with injured, CSF

CSF protein	mean ± SD_ D1 + D2_neg	mean ± SD_D1 + D2_pos	mean_D1 + D2 pos_ D1 + D2 neg	P_D1 + D2 neg vs. D1 + D2 pos	FDR_adj_ P_D1 + D2 neg vs. D1 + D2 pos
KLF6	0.16 ± 0.2	0.34 ± 0.2	0.18	0.0227	0.8779

D0: preoperatively; D1 + D2: postoperative day 1 and postoperative day 2 combined; neg: no delirium; pos: delirium; FDR: false discovery rate

#### Patients with Postoperative Hallucinations

For hallucinations, two proteins differed in serum of affected compared to non-affected patients at uncorrected *p* < 0.01 (NF1 and MGMT) and 11 at *p* < 0.05 (Fig. 4a, b, Table 8). Only NF1 was still significantly higher in patients with hallucinations after FDR correction (Table 8). Compared to baseline, 16 proteins were significantly different at *p* < 0.01 in the patients with postoperative hallucinations, while 26 differed at *p* < 0.05 (Table S5c).

**Table 8:** Postoperative levels in patients with and without postoperative hallucinations, serum

Serum_protein	mean ± SD_D1 + D2_neg	mean ± SD_D1 + D2_pos	mean_D1 + D2 pos_D1 + D2 neg	P_D1 + D2 neg vs. D1 + D2 pos	FDR_adj_ P_D1 + D2 neg vs. D1 + D2 pos
NF1	4.23 ± 1.2	6.01 ± 1.3	1.78	0.0001	0.0059
MGMT	4.29 ± 1.4	5.44 ± 0.8	1.15	0.0025	0.0832
SLIT2	0.21 ± 0.3	0.38 ± 0.3	0.16	0.0108	0.1833
CAMK2G	0.07 ± 0.1	0.17 ± 0.1	0.10	0.0111	0.1833
CSPG4	0.58 ± 0.3	0.76 ± 0.2	0.19	0.0169	0.2227
VIM	5.57 ± 0.7	6.06 ± 0.6	0.49	0.0219	0.2244
SFRP2	2.65 ± 0.8	3.26 ± 0.6	0.61	0.0238	0.2244
NOTCH4	0.4 ± 0.2	0.7 ± 0.4	0.29	0.0417	0.2533
FZD2	0.28 ± 0.3	0.53 ± 0.4	0.25	0.0423	0.2533
FN1	0.78 ± 0.4	0.5 ± 0.4	− 0.28	0.0459	0.2533
MMP2	6.09 ± 0.2	6.22 ± 0.2	0.13	0.0485	0.2533

D0: preoperatively; D1 + D2: postoperative day 1 and postoperative day 2 combined; neg: no hallucinations; pos: hallucinations; FDR: false discovery rate

Only the levels of CC2 differed in CSF between patients with and without postoperative hallucinations at uncorrected *p* < 0.01 and 11 proteins at *p* < 0.05, but none were significant after FDR correction (Fig. 4c, Table 9). IL6, VIM, GFAP, and CHI3L1 differed in the patients with hallucinations after surgery compared to preoperatively (Table S5d), and 14 differed in the patients without postoperative hallucinations compared to preoperatively, after FDR correction (Table S5b). These patients are further described in the supplementary data.

**Table 9:** Postoperative levels in patients with and without postoperative hallucinations, CSF

CSF_Protein	mean ± SD_D1 + D2_neg	mean ± SD_D1 + D2_pos	mean_D1 + D2 pos_D1 + D2 neg	P_D1 + D2 neg vs. D1 + D2 pos	FDR_adj_ P_D1 + D2 neg vs. D1 + D2 pos
CC2	0.15 ± 0.2	0.04 ± 0.1	− 0.11	0.0082	0.2835
IL1B	0.43 ± 1.2	0 ± 0	− 0.43	0.0130	0.2835
NOTCH4	0.12 ± 0.1	0.04 ± 0.1	− 0.08	0.0137	0.2835
BMI1	0.17 ± 0.1	0.08 ± 0.1	− 0.08	0.0180	0.2835
FZD4	0.66 ± 0.2	0.48 ± 0.2	− 0.18	0.0246	0.2835
ITGA2	0.51 ± 0.2	0.37 ± 0.2	− 0.14	0.0310	0.2835
PXN	0.81 ± 0.7	0.35 ± 0.4	− 0.46	0.0341	0.2835
MGMT	1.52 ± 1	0.87 ± 0.6	− 0.65	0.0387	0.2835
BMPR1B	0.51 ± 0.2	0.34 ± 0.2	− 0.17	0.0387	0.2835
TWIST2	0.37 ± 0.3	0.21 ± 0.3	− 0.16	0.0446	0.2872
GAD2	1.02 ± 0.8	0.58 ± 0.3	− 0.45	0.0479	0.2872

D0: preoperatively; D1 + D2: postoperative day 1 and postoperative day 2 combined; neg: no hallucinations; pos: hallucinations; FDR: false discovery rate

## Discussion

In the current study, we present clinical and biochemical data from a cohort operated for complex disease in the thoracic aorta where the surgery entails substantial risks for neurological complications. To our knowledge, the study is the first that describes an extensive protein analysis of both serum and CSF. The findings can aid to identify specific biomarkers of neurologic injury in this patient category.

### Clinical Results

Neurological complications of any sort were common in our material (61%), but the spectrum of severity was wide, where SCI is the most devastating. SCI related to the surgery occurred in 8.7% in our material, which is comparable with incidences in other studies [13]. Other comorbidities, such as atrial fibrillation and delirium, were frequent.

### Biomarker Profiling—General Results of Surgery

There were large differences in the postoperative compared to preoperative protein profiles in both serum and CSF. This is not surprising as the surgery itself triggers a systemic response. Some markers, like IL6 were strongly increased in both serum and CSF postoperatively, whereas others, like VIM showed different patterns in CSF (decreased) and in serum (increased) postoperatively. No further analysis was performed on the samples in the group as a whole, as was on the patient group with neurological complications.

### Specific Analysis of the Patients with Neurological Complications

Biomarkers after traumatic spinal cord injury are fairly well developed [2, 12]. However, biomarkers for SCI after aortic surgery are not as well-established, although some exist, where rises in intrathecal lactate is an early sign of spinal cord ischemia [8, 18]. Also, S100b increases intrathecally after spinal cord ischemia, but this takes longer, up to 6 h [18]. However, increases in S100b cannot distinguish between spinal cord or brain injuries [27].

IL6 is a proinflammatory cytokine, abundant in most organ systems, and GFAP (glial fibrillary acidic protein) is a structural protein in astrocytes. They both exhibited the same trend as the levels were increased postoperatively in all patients, but more so in SCI patients. This makes both IL6 and GFAP less useful as specific markers for SCI, although perhaps a threshold for increased risk of negative outcome could be identified. Increased levels of IL6 in blood have been linked to worse outcome after stroke [6], but the authors conclude, as we do, that the clinical usefulness may be small, since this again may be a question of cutoff levels.

In another study of open thoraco-abdominal aortic aneurysm (TAAA) surgery increased GFAP levels postoperatively were associated with delayed onset of paraplegia [35]. But the conclusion was that the usefulness of biomarkers in predicting SCI was limited, as the levels rose only after the injury had occurred.

It is worth mentioning that there are several interesting proteins that displayed alterations in the injured population compared to the non-injured, but failed to reach statistical significance, in the limited number of samples. CX3CR1 a chemokine receptor on microglia, [14], initially excluded from the analysis (see methods), showed a strong tendency of increase in the CSF of the SCI patients compared to the non-injured postoperatively (*p* = 0.14). This is interesting as blocking of CX3CR1 signaling leads to improved outcome after traumatic SCI in mice [14]. CSPG4 showed increases in serum of the injured compared to the non-injured postoperatively (*p* = 0.027 unadjusted and *p* = 0.14 FDR adjusted) and may turn out to be a candidate in future studies. CSPG4 (chondroitin sulphate proteoglycan 4, also called NG2) is a part of the extracellular matrix and has been studied in the context of traumatic SCI [5]. NG2/CSPG4 is believed to be detrimental and disturb axonal regeneration after traumatic SCI, as inhibition of NG2 with antibodies leads to increased axonal regeneration [31].

In a mixed cardiac surgery population, about 25% suffer postoperative delirium although the range is wide, from 10 to 80% in different settings [16]. Delirium is a potentially severe complication in itself and often necessitates extended ICU-care, but is also a risk for other complications, such as sepsis and sternal instability [16]. Delirium is also associated with long-term cognitive decline and mortality [9]. With respect to aortic surgery, circulatory arrest time influences delirium development negatively and hypothermia, although protective in a general brain perspective, also increases the risk for delirium [16].

Protein biomarkers for delirium after cardiac surgery have been studied to some extent, but no marker has emerged as optimal with regard to specificity or predictive value. Hermann et al. assessed serum levels of NSE and S100b after cardiac surgery and found that increased levels accurately predicted both postoperative delirium and also neurobehavioral outcome up to 6 months after surgery [15]. In line with this, low levels of S100b rule out delirium, although in an off-pump CABG setting [1]. However, neither S100b nor NSE is specific for delirium.

In CSF, KLF6 (Kruppel-like factor-6) was elevated in patients with delirium. KLF6 is a transcriptional activator involved in oligodendrocyte development and myelination processes [19] as well as in axonal growth [30], and levels of KLF6 are modified by miRNA after nerve injury in mice [21].

Both TR4 and EZH2 were elevated in serum in the patients with postoperative delirium; however, the patients that developed postoperative delirium had elevated levels of both TR4 and EZH2 already preoperatively. TR4 and EZH2 could therefore be interesting candidates to predict risk of developing postoperative delirium. EZH2 (enhancer of zeste homolog 2) has been linked to neurodegenerative disorders [20] as well as to learning and memory, thus linking it to cognition [37]. TR4 (testicular receptor 4) has a role in myelination and oligodendrocyte maturation [36] and maintenance of spinal interneurons [33]. The physiological link between TR4 and delirium is not evident, but disturbed interneuron signaling leads to distorted sensory input [36].

Hallucinations may be a part of the delirium state, but also manifests in patients that are otherwise orientated. Apart from being highly unpleasant, it sometimes needs to be treated with neuroleptics, which are not without side effects. NF1 (neurofibromin 1), associated with neurofibromatosis type 1, has been linked to cognitive impairments [24], differed in the patients with and without hallucinations. The fact that there were discrete markers that differed between patients with delirium and hallucinations supports the notion that these are two different cognitive disturbances.

Postspinal puncture headache is a relatively minor complication in the setting of extensive aortic surgery. Nonetheless, it commonly occurs in almost one of five TAAA patients with spinal drain [26], and it can lead to significant morbidity, which often necessitates active treatment [26]. It would be of interest to verify the identified markers further in other spinal headache populations.

An optimal biomarker should be both sensitive and specific. In our study, this would mean that levels should be altered in the injured group compared to the non-injured group postoperatively (D1 + D2_pos vs. D1 + D2_neg), but at the same time not altered between the preoperative group compared to the non-injured postoperative group. Serum CSPG4 and MGMT, as well as CSF CX3CR1, are such candidates in SCI, but after adjustment, the statistical significance disappeared in the limited patient material investigated herein.

Ideally, the results of the current study can contribute to improved, early diagnostics, and consequent treatment of neurological complications after thoracic aortic surgery, which would be of great clinical relevance. Especially prevention of manifest SCI would be of outmost value, as ischemic spinal injuries show poor prognosis with regard to recovery in spite of long rehabilitation [22]. Before clinical application of the current results further confirmation is warranted, especially for the small SCI group. However, the results already suggest that TR4 and EZH2 levels could be used to preoperatively identify patients that are at risk of developing postoperative delirium. For a summary of the markers of greatest interest, see Table 10.

**Table 10:** Summary of most interesting proteins

Protein	Condition	Tissue/fluid	Status in affected	Function	Usefulness in current setting
IL6	SCI	CSF and Serum	Increased	Pleiotropic cytokine, wide range of effects	Unspecific in this setting, perhaps cut-off levels exist?
GFAP	SCI	CSF and Serum	Increased	Structural protein in astrocytes	Unspecific in this setting, perhaps cut-off levels exist?
CSPG4	SCI	Serum	Increased*	Matrix growth, disturbed axonal regeneration	Neutralizing antibodies exist
CX3CR1	SCI	CSF	Increased#	Chemokine receptor on microglia	Deletion improves outcome after SCI in mice
KLF6	Delirium	CSF	Increased	Myelination, axonal growth, glioblastomas	Levels modifiable with miRNA?
TR4	Delirium	Serum	Increased	Interneuron maintenance, oligodendrocyte maturation	Predict development of delirium?
EZH2	Delirium	Serum	Increased	Neural progenitor development, memory and learning	Predict development of delirium?
NF1	Hallucinations	Serum	Increased	Neurofibromatosis, linked to cognitive impairments	Improved diagnostics and disease understanding?

*Not significant after FDR adjustment (*p* = 0.012 unadjusted and *p* = 0.10 FDR adjusted)

#Not significant, *p* = 0.14

When interpreting biomarkers, it is also important to remember the basic pathophysiology and not over-interpret potential variances in protein levels as causative mechanisms. For instance in the setting of SCI, as compared to perhaps delirium and hallucinations, there is most often a distinct surgical/mechanic explanation, with disturbed blood-flow as a causative mechanism. Delirium and hallucinations may manifest their causation more at the microscopic/neurochemical and perhaps individual level. Nonetheless, early postoperative detection would be of large value for all aforementioned conditions.

Serum samples are more easily available than CSF samples and for that reason preferable as biomarkers. However, CSF samples could provide further information and may mirror the CNS compartment better than serum, potentially reflecting pathology earlier. However, this cannot be concluded from our studies, as no CSF samples were available from earlier than the first postoperative morning, and at this time, many changes were already detectable in serum. Perhaps CSF samples taken perioperatively or in the first hours postoperatively could be more sensitive in identifying neurological alterations, and this may be worth exploring further.

A related question concerns the blood-brain barrier (BBB). It is unclear how intact the BBB is after complex aortic surgery, with massive inflammation, extra-corporeal circulation, and CNS ischemia. This is poorly investigated, but a small study suggests BBB disruption even after more standard cardiac surgery like aortic valve replacement [25], which in turn makes it difficult to define the direction of flow between the compartments. There were multiple changes in the CSF levels of all operated patients, even the non-neurologically affected, which is interesting in itself. Further studies could perhaps link CSF alterations to later complications such as cognitive decline and decreased overall function [9, 10].

The study has some limitations, as for instance, the group with SCI is small. Also, two TEVAR patients are included; however, their postoperative samples were excluded so that potential effects of the ECC would be similar in all analyzed samples. Also, there is a possibility that subclinical strokes were missed [29], as neuroimaging was not performed routinely, only upon clinical suspicion.

## Conclusion

Several of the proteins differed between the neurologically affected and non-affected groups, and this is a source for further exploration and verification. However, it is not possible to conclude whether the alterations reflect intrinsic compensatory mechanisms, mediators of subsequent injury, and therefore potential target proteins for treatment or merely markers of damaged tissue. Even though the current study identifies new interesting markers, it needs confirmation and should ideally be complemented with mechanistic experiments.

## Electronic Supplementary Material

ESM 1 (DOCX 799 kb)

## Abbreviations

FET: frozen elephant trunk
POD: postoperative day
SCI: spinal cord injury
TAAA: thoraco-abdominal aortic aneurysm
TEVAR: thoracic endovascular aortic repair
