# Supplementary material for: Protein Profiling in Serum and Cerebrospinal Fluid Following Complex Surgery on the Thoracic Aorta Identifies Biological Markers of Neurologic Injury
Source: J Cardiovasc Transl Res. 2018 Oct 26;11(6):503–16. doi: 10.1007/s12265-018-9835-8 (PMC6294830; doi:10.1007/s12265-018-9835-8)
Supplement: Supplementary file 1 — (DOCX 799 kb) [file 12265_2018_9835_MOESM1_ESM.docx]

# Supplemental Material

**Journal**: Journal of Cardiovascular Translational Research

**Title:** Protein profiling in serum and cerebrospinal fluid following complex surgery on the thoracic aorta identifies biological markers of neurologic injury

**Short title:** Neurology and biomarkers after aortic surgery

**Authors:** Rickard P.F. Lindblom^1, 2*^, Qiujin Shen^3*^, Sofie Axén^1^, Ulf Landegren^3^, Masood Kamali-Moghaddam^3^, Stefan Thelin^1, 2^

*) Equal contribution

1) Department of Cardiothoracic Surgery and Anesthesia, Uppsala University Hospital

2) Department of Surgical Sciences, Section of Thoracic Surgery, Uppsala University

3) Department of Immunology, Genetics and Pathology, Science for Life Laboratory, Uppsala University

**Corresponding author:**

Rickard P.F. Lindblom, e-mail: Rickard.lindblom@surgsci.uu.se.

## Methods

Clinical data

Preoperative data included medical history of hypertension, COPD, hyperlipidemia, diabetes, smoking history, degree of heart failure (NYHA classification), patient BMI, kidney function and previous cardiac surgery. Perioperative variables included indication/diagnose, type of procedure, whether preoperative deviation of left subclavian artery was performed, presence of spinal drain, dimension of aneurysm, and if concomitant surgery was performed. Postoperative variables assessed included mortality, neurological complication- classified as stroke, SCI, delirium/severe confusion, hallucinations or post-puncture spinal headache. The cases with SCI all had a permanent/persisting loss of neurological function where the lesion anatomically could be located to the spinal level. Also frequencies of re-operation for bleeding or infection, pericardiocentesis, pleural drainage, atrial fibrillation and need for dialysis were quantified. See table 1-3 for demographics and baseline values of the study population.

Sample preparation

Both serum and cerebrospinal fluid (CSF) samples were obtained preoperatively and at 8 am in the first and second postoperative mornings. 5ml of whole blood was taken from the arterial line into sample tubes and centrifuged at 4000 rpm at 4^○^C for 10 min, thereafter the serum was aliquoted into smaller sterilized and RNase free tubes and stored at -70^○^C until analysis. 3-4 ml CSF was obtained preoperatively at the time of placing the spinal drainage and aliquoted into the same type of tubes as the serum aliquots. 3-4 ml CSF was then drawn in the postoperative mornings from the spinal drainage after discarding the first few ml and aliquoted. All CSF samples were frozen shortly after collection and stored at -70^○^C until analysis.

Protein measurements

The levels of 92 proteins with relevance to neurology were measured simultaneously by multiplex proximity extension assay (PEA) with only 1 µl for each sample. The assay was performed according to manufactures recommendations ([www.olink.com](http://www.olink.com)). Briefly, samples were incubated overnight at 2-8°C with a set of paired oligonucleotides-conjugated antibodies where two antibodies recognize the same target. The proximity binding brings the paired conjugated oligonucleotides close to each other, allowing them to be hybridized and extended. The extended double-strand DNA can be quantified by qPCR with a microfluidic based PCR system (Fluidigm). Negative control and interplate control were added in to calculate the relative quantification as normalized protein expression (NPX) values. NPX is a unit in log2 scale, which is positively correlated to protein concentrations. Limits of detection (LOD) were calculated for each protein, and defined as protein concentrations at NPX values three standard deviations above the background. All tested proteins are listed in Supplementary table 1.

## Results

There were large differences in the protein profiles in both serum and CSF of all patients, not only the ones that suffered from neurological complicatons, preoperatively compared to postoperatively compared. The most pronounced differences are described below.

Proteins in serum of the whole operated population

For the whole study population, comparing serum levels preoperatively and postoperative day 1 (POD1), levels of 35 proteins differed with a significance at p<0.05, 11 at p<0.01 and 6 at p<0.001 in a non-parametric Mann-Whitney U-test with FDR corrected p-values. Comparing preoperative levels and postoperative day 2 (POD2) 31 proteins differed at p<0.05, 16 at p<0.01 and 7 at p<0.001. The levels of most proteins were altered after operation. The top five significantly changed serum proteins were IL6 (increased), CHI3L1 (decreased), SFRP2 (decreased), RTN4R (decreased), and MDGA1 (decreased) at both POD1 and POD2 compared with preoperative levels (**Fig. 1a-e**). The mean±SD of each protein in preoperative and postoperative day 1 and 2 and p-values are listed in Table S2A. No proteins differed significantly between postoperative days 1 and 2 (Table S2A).

Proteins in cerebrospinal fluid of the whole operated population

For the entire study population, only IL6 differed in CSF with significance at FDR<5% between preoperative levels and POD1 levels. The corresponding differences between preoperative levels and POD2 were 11 proteins at p<0.05, 4 at p<0.01 and 1 at p<0.001. The top five CSF proteins with significantly changed levels at POD2 were IL6 (increased), GFAP (increased), CHI3L1 (decreased), CX3CL1 (increased) and CSPG4 (increased) compared to preoperative levels (**Fig. 1a, b, f-h**). No protein levels differed significantly between postoperative day 1 and day 2. The mean±SD and p-values of all proteins are listed in Table S2B.

Patients with postoperative spinal headache

For post-spinal headache, levels of 4 proteins (SFRP2, SMAD9, TR4, ITGA2) differed in serum between patients with and without post-spinal headache at uncorrected p<0.01 and 16 at p<0.05 between the affected and non-affected group (Table S6A). Only SFRP2 levels were still significantly lower in patients with post-spinal headache after p-values corrected by FDR (**Fig. S3a**, Table S6A). The levels of 16 proteins were significantly different at p<0.01 compared to the baseline (only IL6 increased and all others decreased), while the levels of 29 were different at p<0.05 (Table S6E) in patients that developed postoperative headache compared to preoperatively. The levels of RTN4R, IL1B, and WIF1 differed significantly in CSF postoperatively between patients with and without post-spinal headache at FDR corrected p<0.05 (**Fig. S3b-d**, Table S6B).

## Supplementary Figures

**Supplementary figure 1.** PCA plot (A) and heat-map (B) illustrating an overview of the distribution of data. As expected, the serum and CSF samples were highly differing.


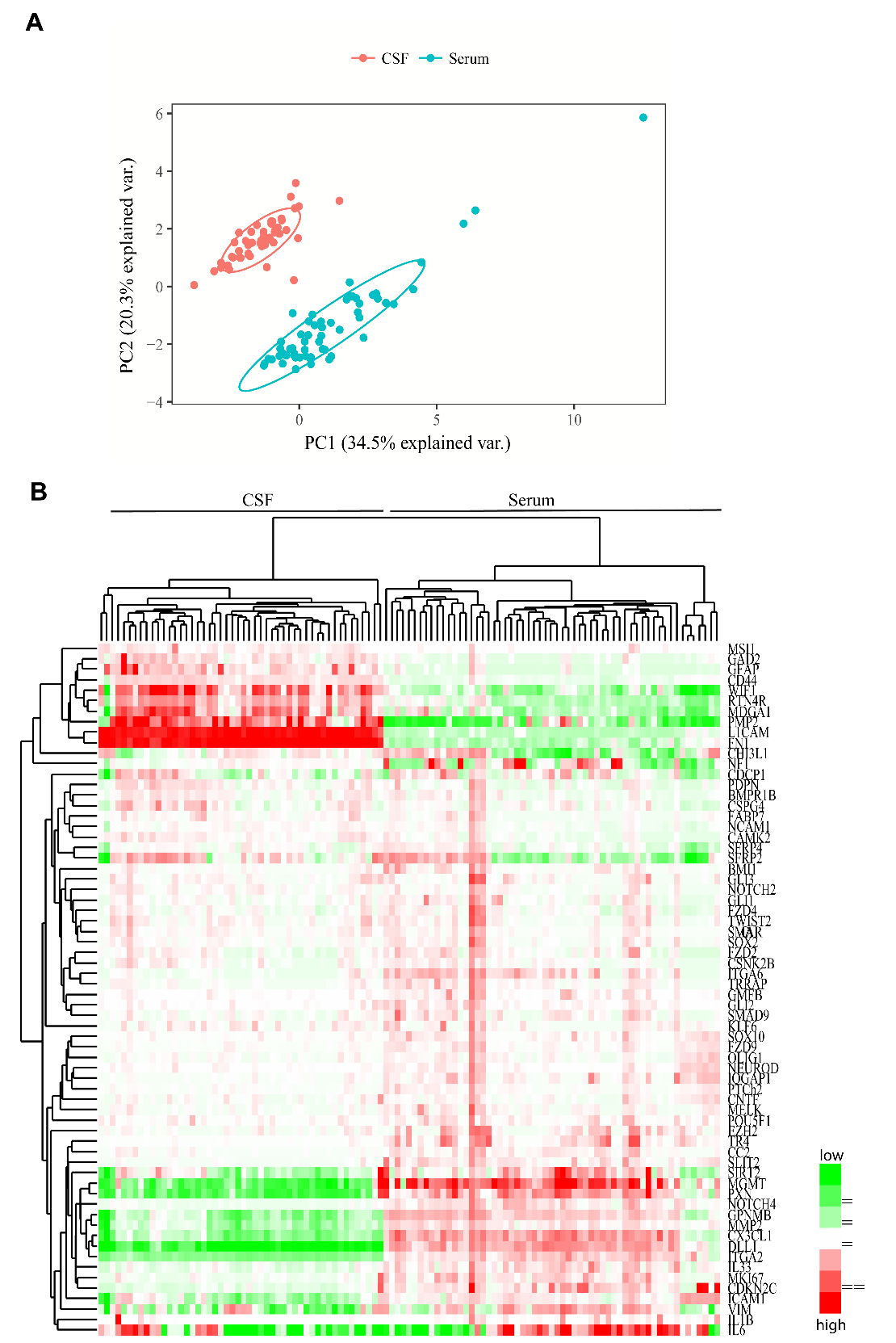


**Supplementary figure 2**

**Supplementary figure 2.** CX3CR1 was in the initial screening of the proteins excluded as the levels in general were very low and below the detection limit of the assay in the majority of the samples. However, a detailed analysis of CX3CR1 in the CSF of patients that suffered postoperative SCI showed a strong trend towards being elevated compared to the patients that did not suffer postoperative SCI (p=0.14). NPX S/N: normalized expression as explained in methods. D0_neg= preoperative levels in patients with no postoperative injury, D0_pos= preoperative levels in patients post-operatively injured, D1+D2_neg = patients that did not suffer SCI postoperatively. D1+D2_pos = patients that suffered SCI postoperatively. N= CSF: D0_neg:18, D0_pos:2, D1+D2_neg:26, D1+D2_pos:4, Serum n= D0_neg:21, D0_pos:1, D1+D2_neg:35, D1+D2_pos:2.

**Supplementary figure 3.**





**Supplementary figure 3.** The proteins differing most significantly in serum (A-D) and CSF (E-H) in the patients that suffered postoperative headache compared to those that did not postoperatively. NPX S/N: normalized expression as explained in methods. D0_neg= preoperative levels in patients with no post-operative no headache, D0_pos= preoperative levels in patients with with post-operative headache, D1+D2_neg = all patients that did not develop headache postoperatively. D1+D2_pos = all patients that developed headache postoperatively. N= CSF: D0_neg:17, D0_pos:3, D1+D2_neg:24, D1+D2_pos:6, Serum n= D0_neg:18, D0_pos:4, D1+D2_neg:29, D1+D2_pos:8.

## Supplementary Tables

**Supplementary table 1.** List of all proteins tested in the study.

| **Short name** | **Full name** | **UniProt ID** | **%^†^ in all** | **%^†^ in CSF** | **%^†^ in serum** | **Excluded** |
| --- | --- | --- | --- | --- | --- | --- |
| BMI1 | Polycomb complex protein BMI-1 | P35226 | 37.9% | 44.0% | 33.3% |  |
| BMP4 | Bone morphogenetic protein 4 | P12644 | NA | NA | NA | yes^a^ |
| BMPR1B | Bone morphogenetic protein receptor type-1B | O00238 | 11.2% | 6.0% | 16.7% |  |
| CAMK2G | Calcium/calmodulin-dependent protein kinase type II subunit gamma | Q13555 | 15.5% | 0.0% | 28.3% |  |
| CCNA2 | Cyclin-A2 | P20248 | 37.9% | 62.0% | 16.7% |  |
| CD44 | CD44 antigen | P16070 | 0.0% | 0.0% | 0.0% |  |
| CDCP1 | CUB domain-containing protein 1 | Q9H5V8 | 0.0% | 0.0% | 0.0% |  |
| CDKN2C | Cyclin-dependent kinase 4 inhibitor C | P42773 | 3.5% | 8.0% | 0.0% |  |
| CHI3L1 | Chitinase-3-like protein 1 | P36222 | 0.0% | 0.0% | 0.0% |  |
| CNTF | Ciliary neurotrophic factor | P26441 | 15.5% | 16.0% | 15.0% |  |
| CSNK2B | Casein kinase II subunit beta | P67870 | 21.6% | 24.0% | 18.3% |  |
| CSPG4 | Chondroitin sulfate proteoglycan 4 | Q6UVK1 | 0.9% | 2.0% | 0.0% |  |
| CX3CL1 | Fractalkine | P78423 | 0.0% | 0.0% | 0.0% |  |
| CX3CR1 | CX3C chemokine receptor 1 | P49238 | 58.6% | 52.0% | 60.0% | yes^b^ |
| DCLK1 | Serine/threonine-protein kinase DCLK1 | O15075 | 78.5% | 98.0% | 63.3% | yes^b^ |
| DLL1 | Delta-like protein 1 | O00548 | 0.0% | 0.0% | 0.0% |  |
| EZH2 | Histone-lysine N-methyltransferase EZH2 | Q15910 | 32.8% | 40.0% | 28.3% |  |
| FABP7 | Fatty acid-binding protein, brain | O15540 | 31.0% | 16.0% | 45.0% |  |
| FN1 | Fibronectin | P02751 | 4.3% | 0.0% | 8.3% |  |
| FZD2 | Frizzled-2 | Q14332 | 12.9% | 6.0% | 16.7% |  |
| FZD4 | Frizzled-4 | Q9ULV1 | 0.0% | 0.0% | 0.0% |  |
| FZD9 | Frizzled-9 | O00144 | 26.7% | 40.0% | 16.7% |  |
| GAD2 | Glutamate decarboxylase 2 | Q05329 | 25.9% | 4.0% | 46.7% |  |
| GFAP | Glial fibrillary acidic protein | P14136 | 30.2% | 0.0% | 53.3% |  |
| GLI1 | Zinc finger protein GLI1 | P08151 | 13.8% | 6.0% | 18.3% |  |
| GLI2 | Zinc finger protein GLI2 | P10070 | 47.4% | 60.0% | 36.7% |  |
| GLI3 | Transcriptional activator GLI3 | P10071 | 34.5% | 28.0% | 41.7% |  |
| GMFB | Glia maturation factor beta | P60983 | 49.1% | 68.0% | 33.3% |  |
| GPNMB | Transmembrane glycoprotein NMB | Q14956 | 0.0% | 0.0% | 0.0% |  |
| HES1 | Transcription factor HES-1 | Q14469 | 81.0% | 88.0% | 75.0% | yes^b^ |
| HOXB1 | Homeobox protein Hox-B1 | P14653 | 93.1% | 96.0% | 88.3% | yes^b^ |
| ICAM1 | Intercellular adhesion molecule 1 | P05362 | 0.0% | 0.0% | 0.0% |  |
| IL1B | Interleukin-1 beta | P01584 | 66.4% | 88.0% | 45.0% |  |
| IL33 | Interleukin-33 | O95760 | 6.0% | 12.0% | 1.7% |  |
| IL6 | Interleukin-6 | P05231 | 0.0% | 0.0% | 0.0% |  |
| IQGAP1 | Ras GTPase-activating-like protein IQGAP1 | P46940 | 34.5% | 52.0% | 21.7% |  |
| ITGA2 | Integrin alpha-2 | P17301 | 0.0% | 0.0% | 0.0% |  |
| ITGA6 | Integrin alpha-6 | P23229 | 32.8% | 52.0% | 18.3% |  |
| JAG1 | Protein jagged-1 | P78504 | 76.7% | 86.0% | 71.7% | yes^b^ |
| KLF4 | Krueppel-like factor 4 | O43474 | 82.8% | 90.0% | 76.7% | yes^b^ |
| KLF6 | Krueppel-like factor 6 | Q99612 | 34.5% | 30.0% | 41.7% |  |
| L1CAM | Neural cell adhesion molecule L1 | P32004 | 11.2% | 0.0% | 21.7% |  |
| MAPT | Microtubule-associated protein tau | P10636 | 87.1% | 92.0% | 80.0% | yes^b^ |
| MDGA1 | MAM domain-containing glycosylphosphatidylinositol anchor protein 1 | Q8NFP4 | 0.0% | 0.0% | 0.0% |  |
| MEF2C | Myocyte-specific enhancer factor 2C | Q06413 | NA | NA | NA | yes^a^ |
| MELK | Maternal embryonic leucine zipper kinase | Q14680 | 32.8% | 42.0% | 26.7% |  |
| MGMT | Methylated-DNA-protein-cysteine methyltransferase | P16455 | 1.7% | 4.0% | 0.0% |  |
| MKI67 | Antigen KI-67 | P46013 | 21.6% | 40.0% | 6.7% |  |
| MMP2 | 72 kDa type IV collagenase | P08253 | 0.0% | 0.0% | 0.0% |  |
| MSI1 | RNA-binding protein Musashi homolog 1 | O43347 | 56.9% | 24.0% | 81.7% |  |
| MSI2 | RNA-binding protein Musashi homolog 2 | Q96DH6 | 62.9% | 66.0% | 58.3% | yes^b^ |
| NCAM1 | Neural cell adhesion molecule 1 | P13591 | 0.0% | 0.0% | 0.0% |  |
| NES | Nestin | P48681 | 91.4% | 98.0% | 83.3% | yes^b^ |
| NEUROD1 | Neurogenic differentiation factor 1 | Q13562 | 48.3% | 64.0% | 38.3% |  |
| NF1 | Neurofibromin | P21359 | 0.0% | 0.0% | 0.0% |  |
| NGFR | Tumor necrosis factor receptor superfamily member 16 | P08138 | 69.0% | 54.0% | 81.7% | yes^b^ |
| NOG | Noggin | Q13253 | 74.1% | 78.0% | 71.7% | yes^b^ |
| NOTCH2 | Neurogenic locus notch homolog protein 2 | Q04721 | 22.4% | 8.0% | 31.7% |  |
| NOTCH4 | Neurogenic locus notch homolog protein 4 | Q99466 | 26.7% | 56.0% | 1.7% |  |
| OLIG1 | Oligodendrocyte transcription factor 1 | Q8TAK6 | 7.8% | 2.0% | 11.7% |  |
| OLIG2 | Oligodendrocyte transcription factor 2 | Q13516 | 94.8% | 96.0% | 93.3% | yes^b^ |
| PDPN | Podoplanin | Q86YL7 | 14.7% | 6.0% | 21.7% |  |
| PMP2 | Myelin P2 protein | P02689 | 10.3% | 0.0% | 16.7% |  |
| POU5F1 | POU domain, class 5, transcription factor 1 | Q01860 | 47.4% | 62.0% | 36.7% |  |
| PTCH1 | Protein patched homolog 1 | Q13635 | 81.9% | 96.0% | 68.3% | yes^b^ |
| PTCh2 | Protein patched homolog 2 | Q9Y6C5 | 21.6% | 26.0% | 18.3% |  |
| PXN | Paxillin | P49023 | 7.8% | 18.0% | 0.0% |  |
| RTN4R | Reticulon-4 receptor | Q9BZR6 | 0.0% | 0.0% | 0.0% |  |
| RTN4RL1 | Reticulon-4 receptor-like 1 | Q86UN2 | 75.0% | 96.0% | 58.3% | yes^b^ |
| S100B | Protein S100-B | P04271 | 96.6% | 94.0% | 96.7% | yes^b^ |
| SFRP2 | Secreted frizzled-related protein 2 | Q96HF1 | 0.0% | 0.0% | 0.0% |  |
| SFRP4 | Secreted frizzled-related protein 4 | Q6FHJ7 | 0.0% | 0.0% | 0.0% |  |
| SIRT2 | NAD-dependent protein deacetylase sirtuin-2 | Q8IXJ6 | 0.0% | 0.0% | 0.0% |  |
| SLAIN1 | SLAIN motif-containing protein 1 | Q8ND83 | NA | NA | NA | yes^a^ |
| SLC1A3 | Excitatory amino acid transporter 1 | P43003 | 81.0% | 76.0% | 85.0% | yes^b^ |
| SLIT2 | Slit homolog 2 protein | O94813 | 35.3% | 68.0% | 10.0% |  |
| SMAD9 | Mothers against decapentaplegic homolog 9 | O15198 | 13.8% | 12.0% | 16.7% |  |
| SMARCA4 | Transcription activator BRG1 | P51532 | 37.1% | 26.0% | 45.0% |  |
| SOX1 | Transcription factor SOX-1 | O00570 | 74.1% | 92.0% | 58.3% | yes^b^ |
| SOX10 | Transcription factor SOX-10 | P56693 | 45.7% | 58.0% | 35.0% |  |
| SOX11 | Transcription factor SOX-11 | P35716 | 91.4% | 100.0% | 85.0% | yes^b^ |
| SOX2 | Transcription factor SOX-2 | P48431 | 31.0% | 30.0% | 31.7% |  |
| SOX21 | Transcription factor SOX-21 | Q9Y651 | 85.3% | 96.0% | 73.3% | yes^b^ |
| SOX5 | Transcription factor SOX-5 | P35711 | 83.6% | 90.0% | 78.3% | yes^b^ |
| TPH1 | Tryptophan 5-hydroxylase 1 | P17752 | 90.5% | 100.0% | 83.3% | yes^b^ |
| TR4 | Nuclear receptor subfamily 2 group C member 2 | P49116 | 41.4% | 70.0% | 20.0% |  |
| TRRAP | Transformation/transcription domain-associated protein | Q9Y4A5 | 31.0% | 40.0% | 23.3% |  |
| TWIST2 | Twist-related protein 2 | Q8WVJ9 | 25.9% | 24.0% | 28.3% |  |
| VAMP2 | Vesicle-associated membrane protein 2 | P63027 | NA | NA | NA | yes^a^ |
| WIF1 | Wnt inhibitory factor 1 | Q9Y5W5 | 0.0% | 0.0% | 0.0% |  |
| VIM | Vimentin | P08670 | 0.0% | 0.0% | 0.0% |  |
| WISP1 | WNT1-inducible-signaling pathway protein 1 | O95388 | NA | NA | NA | yes^a^ |

†; Percentage of samples with protein level less than limit of detection.

NA: not available due to suspected antibody cross-activity.

yes^a^: 5 proteins excluded due to suspected antibody cross-activity.

yes^b^: 21 proteins excluded due to the percentages of detectability of these proteins were less than 50% in both serum and CSF samples in all the pre- and postoperative groups.

**Supplementary table 2a.** Protein levels and p-values in the whole population in serum preoperatively compared with postoperatively.

| **Protein** | **mean±SD_D0** | **mean±SD_POD1** | **mean±SD_POD2** | **ΔNPX_ POD1-D0** | **ΔNPX_ POD2-D0** | **P_D0 vs. POD1** | **FDR_adj_ P_D0 vs. POD1** | **P_D0 vs. POD2** | **FDR_adj_ P_D0 vs. POD2** | **P_POD1 vs. POD2** | **FDR_adj_ P_POD1 vs. POD2** |
| --- | --- | --- | --- | --- | --- | --- | --- | --- | --- | --- | --- |
| IL6 | 4.04±1.5 | 8.04±1.5 | 8.29±1.2 | 4.00 | 4.25 | 1.59E-09 | 4.89E-08 | 1.18E-09 | 5.65E-08 | 0.4612 | 0.9848 |
| CHI3L1 | 9.97±0.4 | 7.86±0.9 | 8.08±0.9 | -2.10 | -1.88 | 1.55E-10 | 1.03E-08 | 1.71E-09 | 5.65E-08 | 0.3909 | 0.9848 |
| SFRP2 | 4.87±0.6 | 2.79±0.7 | 3.08±0.8 | -2.08 | -1.79 | 2.22E-09 | 4.89E-08 | 2.82E-08 | 6.20E-07 | 0.2100 | 0.9848 |
| RTN4R | 2.5±0.4 | 1.78±0.3 | 1.79±0.3 | -0.73 | -0.71 | 3.93E-06 | 5.18E-05 | 4.94E-06 | 8.15E-05 | 0.7986 | 0.9848 |
| MDGA1 | 4.07±0.7 | 3.43±0.6 | 3.16±0.5 | -0.64 | -0.91 | 0.0181 | 0.0411 | 2.18E-05 | 0.0003 | 0.0805 | 0.7592 |
| SFRP4 | 2.73±0.4 | 2.38±0.4 | 2.24±0.5 | -0.35 | -0.49 | 0.0024 | 0.0120 | 7.10E-05 | 0.0008 | 0.1991 | 0.9848 |
| ITGA2 | 2.02±0.4 | 2.24±0.3 | 2.5±0.3 | 0.22 | 0.48 | 0.0210 | 0.0428 | 9.37E-05 | 0.0009 | 0.0805 | 0.7592 |
| FABP7 | 0.3±0.3 | 0.04±0.1 | 0.06±0.1 | -0.26 | -0.24 | 3.07E-05 | 0.0003 | 0.0002 | 0.0020 | 0.6384 | 0.9848 |
| NCAM1 | 9.06±0.2 | 8.87±0.1 | 8.83±0.1 | -0.19 | -0.23 | 0.0006 | 0.0051 | 0.0003 | 0.0022 | 0.4430 | 0.9848 |
| GPNMB | 6.8±0.4 | 6.46±0.5 | 6.38±0.5 | -0.34 | -0.42 | 0.0014 | 0.0092 | 0.0006 | 0.0041 | 0.4252 | 0.9848 |
| OLIG1 | 0.46±0.3 | 0.26±0.2 | 0.17±0.2 | -0.20 | -0.29 | 0.0207 | 0.0428 | 0.0010 | 0.0063 | 0.2058 | 0.9848 |
| VIM | 4.88±1 | 5.78±0.7 | 5.82±0.6 | 0.90 | 0.94 | 0.0026 | 0.0122 | 0.0012 | 0.0066 | 0.9880 | 0.9880 |
| PMP2 | 0.36±0.5 | 2.33±1.3 | 1.32±1.1 | 1.98 | 0.97 | 5.95E-07 | 9.82E-06 | 0.0016 | 0.0083 | 0.0201 | 0.4905 |
| BMPR1B | 0.42±0.3 | 0.16±0.2 | 0.14±0.2 | -0.26 | -0.28 | 0.0038 | 0.0168 | 0.0018 | 0.0086 | 0.7254 | 0.9848 |
| PTCh2 | 0.34±0.3 | 0.12±0.1 | 0.13±0.1 | -0.22 | -0.21 | 0.0004 | 0.0037 | 0.0022 | 0.0091 | 0.9633 | 0.9880 |
| GLI2 | 0.36±0.4 | 0.12±0.1 | 0.08±0.1 | -0.24 | -0.28 | 0.0463 | 0.0727 | 0.0022 | 0.0091 | 0.1457 | 0.9848 |
| FZD9 | 0.38±0.3 | 0.13±0.1 | 0.15±0.2 | -0.25 | -0.24 | 0.0015 | 0.0092 | 0.0029 | 0.0112 | 0.8423 | 0.9848 |
| ITGA6 | 0.83±0.5 | 0.62±0.5 | 0.39±0.3 | -0.21 | -0.45 | 0.1817 | 0.2352 | 0.0038 | 0.0140 | 0.1194 | 0.9846 |
| MELK | 0.46±0.5 | 0.17±0.2 | 0.14±0.1 | -0.29 | -0.32 | 0.0166 | 0.0404 | 0.0047 | 0.0161 | 0.7209 | 0.9848 |
| MMP2 | 6.37±0.3 | 6.14±0.2 | 6.16±0.2 | -0.23 | -0.21 | 0.0082 | 0.0247 | 0.0049 | 0.0161 | 0.7074 | 0.9848 |
| GFAP | 0.24±0.3 | 0.06±0.1 | 0.09±0.2 | -0.18 | -0.15 | 0.0092 | 0.0254 | 0.0085 | 0.0254 | 0.9401 | 0.9848 |
| NEUROD1 | 0.33±0.3 | 0.11±0.2 | 0.11±0.2 | -0.21 | -0.22 | 0.0047 | 0.0186 | 0.0092 | 0.0254 | 0.6591 | 0.9848 |
| BMI1 | 0.47±0.5 | 0.13±0.2 | 0.13±0.2 | -0.35 | -0.34 | 0.0067 | 0.0209 | 0.0092 | 0.0254 | 0.8866 | 0.9848 |
| TRRAP | 0.5±0.5 | 0.18±0.2 | 0.18±0.2 | -0.32 | -0.32 | 0.0065 | 0.0209 | 0.0095 | 0.0254 | 0.9877 | 0.9880 |
| TWIST2 | 0.47±0.6 | 0.12±0.2 | 0.18±0.3 | -0.35 | -0.29 | 0.0011 | 0.0078 | 0.0098 | 0.0254 | 0.9117 | 0.9848 |
| CSNK2B | 0.37±0.3 | 0.16±0.1 | 0.18±0.1 | -0.21 | -0.20 | 0.0062 | 0.0209 | 0.0100 | 0.0254 | 0.7485 | 0.9848 |
| SOX2 | 0.4±0.5 | 0.1±0.1 | 0.16±0.3 | -0.30 | -0.24 | 0.0048 | 0.0186 | 0.0105 | 0.0257 | 0.5978 | 0.9848 |
| SLIT2 | 0.57±0.4 | 0.29±0.3 | 0.29±0.3 | -0.28 | -0.28 | 0.0060 | 0.0209 | 0.0110 | 0.0260 | 0.9394 | 0.9848 |
| GLI1 | 0.56±0.5 | 0.3±0.3 | 0.28±0.4 | -0.26 | -0.28 | 0.0253 | 0.0476 | 0.0116 | 0.0264 | 0.4720 | 0.9848 |
| SOX10 | 0.36±0.3 | 0.16±0.2 | 0.17±0.3 | -0.20 | -0.19 | 0.0117 | 0.0302 | 0.0123 | 0.0271 | 0.6864 | 0.9848 |
| CAMK2G | 0.32±0.3 | 0.12±0.1 | 0.12±0.2 | -0.20 | -0.20 | 0.0172 | 0.0404 | 0.0188 | 0.0401 | 0.7917 | 0.9848 |
| FZD2 | 0.65±0.4 | 0.41±0.3 | 0.39±0.4 | -0.24 | -0.26 | 0.0328 | 0.0570 | 0.0243 | 0.0502 | 0.8194 | 0.9848 |
| CNTF | 0.35±0.3 | 0.17±0.2 | 0.21±0.3 | -0.17 | -0.13 | 0.0220 | 0.0428 | 0.0485 | 0.0970 | 0.8907 | 0.9848 |
| PXN | 3.15±0.9 | 3.44±0.7 | 3.58±0.8 | 0.29 | 0.43 | 0.1636 | 0.2297 | 0.0546 | 0.1060 | 0.6852 | 0.9848 |
| WIF1 | 5.89±0.9 | 5.82±1 | 5.39±0.9 | -0.07 | -0.50 | 0.7860 | 0.8106 | 0.0621 | 0.1170 | 0.2100 | 0.9848 |
| GMFB | 0.32±0.4 | 0.12±0.2 | 0.17±0.3 | -0.21 | -0.16 | 0.0119 | 0.0302 | 0.0911 | 0.1671 | 0.5758 | 0.9848 |
| GAD2 | 0.22±0.3 | 0.09±0.2 | 0.14±0.2 | -0.13 | -0.07 | 0.0219 | 0.0428 | 0.0974 | 0.1691 | 0.7144 | 0.9848 |
| SMARCA4 | 0.28±0.4 | 0.07±0.1 | 0.1±0.1 | -0.21 | -0.18 | 0.0288 | 0.0514 | 0.0974 | 0.1691 | 0.5293 | 0.9848 |
| SMAD9 | 0.54±0.5 | 0.31±0.3 | 0.3±0.3 | -0.24 | -0.24 | 0.1017 | 0.1492 | 0.1052 | 0.1780 | 0.8907 | 0.9848 |
| IQGAP1 | 0.47±0.4 | 0.28±0.4 | 0.32±0.4 | -0.20 | -0.15 | 0.0447 | 0.0720 | 0.1108 | 0.1828 | 0.7465 | 0.9848 |
| L1CAM | 0.26±0.3 | 0.2±0.3 | 0.21±0.3 | -0.06 | -0.05 | 0.1933 | 0.2453 | 0.1519 | 0.2380 | 0.8663 | 0.9848 |
| FZD4 | 0.84±0.6 | 0.48±0.2 | 0.56±0.3 | -0.36 | -0.29 | 0.0090 | 0.0254 | 0.1551 | 0.2380 | 0.3744 | 0.9848 |
| DLL1 | 10.61±0.6 | 10.97±0.4 | 10.87±0.4 | 0.36 | 0.26 | 0.0446 | 0.0720 | 0.1551 | 0.2380 | 0.4612 | 0.9848 |
| TR4 | 0.7±0.7 | 0.4±0.6 | 0.4±0.6 | -0.29 | -0.30 | 0.1771 | 0.2352 | 0.1723 | 0.2585 | 0.8423 | 0.9848 |
| EZH2 | 0.66±0.7 | 0.44±0.4 | 0.34±0.5 | -0.22 | -0.33 | 0.4853 | 0.5510 | 0.1889 | 0.2770 | 0.3023 | 0.9848 |
| POU5F1 | 0.31±0.4 | 0.19±0.3 | 0.18±0.3 | -0.12 | -0.13 | 0.2003 | 0.2495 | 0.1940 | 0.2784 | 0.6942 | 0.9848 |
| NOTCH2 | 0.27±0.3 | 0.11±0.2 | 0.15±0.2 | -0.16 | -0.12 | 0.0442 | 0.0720 | 0.2176 | 0.3055 | 0.5471 | 0.9848 |
| KLF6 | 0.39±0.5 | 0.13±0.2 | 0.17±0.2 | -0.26 | -0.22 | 0.1624 | 0.2297 | 0.2551 | 0.3508 | 0.7131 | 0.9848 |
| SIRT2 | 3.73±0.8 | 4.64±1 | 4.09±1.1 | 0.91 | 0.36 | 0.0019 | 0.0107 | 0.2625 | 0.3535 | 0.0310 | 0.5111 |
| CDCP1 | 4.1±0.8 | 4.14±0.7 | 3.87±0.7 | 0.04 | -0.23 | 0.9897 | 0.9897 | 0.2742 | 0.3620 | 0.1785 | 0.9848 |
| CC2 | 0.32±0.4 | 0.28±0.3 | 0.22±0.3 | -0.04 | -0.10 | 0.9896 | 0.9897 | 0.3198 | 0.4138 | 0.3596 | 0.9848 |
| PDPN | 0.45±0.4 | 0.2±0.3 | 0.29±0.3 | -0.25 | -0.16 | 0.0187 | 0.0411 | 0.3616 | 0.4590 | 0.3023 | 0.9848 |
| IL1B | 0.17±0.4 | 0.25±0.4 | 0.12±0.2 | 0.08 | -0.05 | 0.4565 | 0.5286 | 0.3691 | 0.4596 | 0.9124 | 0.9848 |
| MGMT | 4.57±1.2 | 5.23±1.2 | 4.38±1.3 | 0.67 | -0.19 | 0.0731 | 0.1096 | 0.4590 | 0.5610 | 0.0223 | 0.4905 |
| CDKN2C | 1.17±1.4 | 1.22±0.9 | 1.12±0.8 | 0.05 | -0.05 | 0.1717 | 0.2352 | 0.5449 | 0.6421 | 0.5990 | 0.9848 |
| CD44 | 5.78±0.2 | 5.77±0.1 | 5.77±0.1 | -0.01 | -0.01 | 0.7076 | 0.7413 | 0.5449 | 0.6421 | 0.7986 | 0.9848 |
| CX3CL1 | 5.29±0.7 | 5.63±0.6 | 5.2±0.6 | 0.35 | -0.09 | 0.0611 | 0.0937 | 0.5999 | 0.6826 | 0.0050 | 0.3284 |
| ICAM1 | 10.79±0.4 | 10.76±0.4 | 10.72±0.4 | -0.04 | -0.07 | 0.7076 | 0.7413 | 0.5999 | 0.6826 | 0.8219 | 0.9848 |
| FN1 | 0.56±0.4 | 0.71±0.5 | 0.59±0.4 | 0.16 | 0.03 | 0.2664 | 0.3256 | 0.6341 | 0.7093 | 0.4565 | 0.9848 |
| CSPG4 | 0.73±0.3 | 0.63±0.2 | 0.69±0.3 | -0.09 | -0.03 | 0.4925 | 0.5510 | 0.7166 | 0.7883 | 0.7074 | 0.9848 |
| MSI1 | 0.04±0.1 | 0.04±0.2 | 0.04±0.1 | 0.00 | 0.00 | 0.5659 | 0.6123 | 0.7408 | 0.8016 | 0.3812 | 0.9848 |
| IL33 | 0.79±0.4 | 1.05±0.4 | 0.79±0.5 | 0.26 | 0.00 | 0.0279 | 0.0512 | 0.8402 | 0.8882 | 0.0656 | 0.7592 |
| GLI3 | 0.4±0.7 | 0.14±0.2 | 0.27±0.4 | -0.26 | -0.13 | 0.1794 | 0.2352 | 0.8559 | 0.8882 | 0.3407 | 0.9848 |
| NF1 | 4.8±1.6 | 5.22±1.5 | 4.86±1.6 | 0.42 | 0.06 | 0.3178 | 0.3814 | 0.8613 | 0.8882 | 0.4252 | 0.9848 |
| MKI67 | 0.65±0.5 | 0.48±0.3 | 0.64±0.5 | -0.18 | -0.01 | 0.4101 | 0.4834 | 0.8811 | 0.8947 | 0.2875 | 0.9848 |
| NOTCH4 | 0.57±0.4 | 0.53±0.4 | 0.55±0.3 | -0.04 | -0.02 | 0.5091 | 0.5600 | 0.9678 | 0.9678 | 0.8689 | 0.9848 |

D0: preoperatively, POD1; postoperative day 1, POD2: postoperative day 2.

**Supplementary table 2b.** Protein levels and p-values in the whole population in CSF preoperatively compared with postoperatively.

| **Protein** | **mean±SD_D0** | **mean±SD_POD1** | **mean±SD_POD2** | **ΔNPX_ POD1-D0** | **ΔNPX_**  **POD2-D0** | **P_D0 vs. POD1** | **FDR_adj_ P_D0 vs. POD1** | **P_D0 vs. POD2** | **FDR_adj_ P_D0 vs. POD2** | **P_POD1 vs. POD2** | **FDR_adj_ P_POD1 vs. POD2** |
| --- | --- | --- | --- | --- | --- | --- | --- | --- | --- | --- | --- |
| IL6 | 2.69±1.2 | 6.37±1.5 | 6.28±1.7 | 3.68 | 3.59 | 4.21E-07 | 2.78E-05 | 8.56E-08 | 5.65E-06 | 0.838 | 0.982 |
| GFAP | 0.65±0.3 | 0.99±0.5 | 1.86±1.4 | 0.33 | 1.21 | 0.019 | 0.252 | 3.62E-05 | 0.001 | 0.029 | 0.662 |
| CHI3L1 | 9.49±0.2 | 9.34±0.3 | 8.78±0.7 | -0.15 | -0.71 | 0.064 | 0.35 | 6.34E-05 | 0.001 | 0.001 | 0.093 |
| CX3CL1 | 2.96±0.4 | 3.41±0.5 | 3.59±1 | 0.45 | 0.63 | 0.007 | 0.226 | 0.001 | 0.01 | 0.137 | 0.668 |
| CSPG4 | 0.62±0.3 | 0.84±0.4 | 1.13±0.6 | 0.22 | 0.51 | 0.069 | 0.35 | 0.002 | 0.022 | 0.148 | 0.668 |
| VIM | 5.08±1.2 | 4.11±1 | 3.73±1.2 | -0.97 | -1.35 | 0.014 | 0.252 | 0.003 | 0.022 | 0.412 | 0.851 |
| ICAM1 | 9.44±0.7 | 9.81±0.6 | 10.15±1.1 | 0.37 | 0.71 | 0.059 | 0.35 | 0.003 | 0.022 | 0.056 | 0.668 |
| CSNK2B | 0.14±0.2 | 0.22±0.2 | 0.38±0.2 | 0.08 | 0.25 | 0.141 | 0.441 | 0.003 | 0.022 | 0.065 | 0.668 |
| SFRP4 | 2.6±0.2 | 2.52±0.2 | 2.35±0.4 | -0.08 | -0.25 | 0.074 | 0.35 | 0.005 | 0.036 | 0.161 | 0.668 |
| ITGA2 | 0.27±0.1 | 0.4±0.2 | 0.46±0.3 | 0.13 | 0.19 | 0.019 | 0.252 | 0.008 | 0.046 | 0.567 | 0.935 |
| DLL1 | 6.35±0.9 | 6.89±0.7 | 7.24±1.5 | 0.54 | 0.89 | 0.043 | 0.35 | 0.008 | 0.046 | 0.202 | 0.668 |
| GLI2 | 0.11±0.2 | 0.04±0.1 | 0.03±0.1 | -0.07 | -0.08 | 0.33 | 0.673 | 0.011 | 0.06 | 0.174 | 0.668 |
| PDPN | 0.43±0.2 | 0.53±0.3 | 0.7±0.3 | 0.10 | 0.27 | 0.211 | 0.516 | 0.012 | 0.06 | 0.098 | 0.668 |
| IL1B | 0±0 | 0.04±0.1 | 0.31±1.1 | 0.04 | 0.31 | 0.106 | 0.438 | 0.017 | 0.077 | 0.358 | 0.793 |
| FABP7 | 0.14±0.2 | 0.21±0.2 | 0.32±0.3 | 0.07 | 0.18 | 0.165 | 0.466 | 0.018 | 0.077 | 0.372 | 0.793 |
| FZD2 | 0.27±0.2 | 0.39±0.2 | 0.48±0.3 | 0.11 | 0.20 | 0.092 | 0.406 | 0.019 | 0.077 | 0.345 | 0.793 |
| FN1 | 4.9±0.6 | 4.62±0.4 | 4.4±0.7 | -0.28 | -0.50 | 0.131 | 0.441 | 0.03 | 0.117 | 0.187 | 0.668 |
| SOX2 | 0.08±0.1 | 0.15±0.1 | 0.19±0.2 | 0.08 | 0.11 | 0.031 | 0.338 | 0.038 | 0.136 | 0.868 | 0.982 |
| GPNMB | 4.71±0.7 | 5.11±0.6 | 5.12±1.1 | 0.40 | 0.41 | 0.064 | 0.35 | 0.039 | 0.136 | 0.345 | 0.793 |
| CD44 | 6.26±0.2 | 6.31±0.2 | 6.33±0.3 | 0.05 | 0.06 | 0.564 | 0.791 | 0.043 | 0.139 | 0.233 | 0.668 |
| CAMK2G | 0.37±0.2 | 0.41±0.1 | 0.5±0.2 | 0.04 | 0.13 | 0.314 | 0.673 | 0.046 | 0.139 | 0.217 | 0.668 |
| NF1 | 5±0.1 | 4.97±0.1 | 4.82±0.3 | -0.03 | -0.18 | 0.458 | 0.756 | 0.046 | 0.139 | 0.217 | 0.668 |
| MKI67 | 0.17±0.4 | 0.08±0.1 | 0.2±0.2 | -0.08 | 0.03 | 0.658 | 0.804 | 0.057 | 0.163 | 0.03 | 0.662 |
| SIRT2 | 3.22±1.5 | 3.07±0.7 | 3.47±1.1 | -0.15 | 0.25 | 0.657 | 0.804 | 0.074 | 0.201 | 0.116 | 0.668 |
| TRRAP | 0.07±0.1 | 0.1±0.1 | 0.15±0.1 | 0.03 | 0.07 | 0.391 | 0.732 | 0.076 | 0.201 | 0.31 | 0.788 |
| BMPR1B | 0.29±0.2 | 0.39±0.2 | 0.42±0.2 | 0.10 | 0.14 | 0.147 | 0.441 | 0.08 | 0.203 | 0.595 | 0.935 |
| ITGA6 | 0.08±0.2 | 0.11±0.1 | 0.15±0.2 | 0.03 | 0.07 | 0.183 | 0.466 | 0.084 | 0.206 | 0.697 | 0.98 |
| CDCP1 | 3.63±0.7 | 4.03±0.8 | 3.95±1.1 | 0.40 | 0.33 | 0.122 | 0.441 | 0.122 | 0.278 | 0.775 | 0.982 |
| MMP2 | 5.04±0.6 | 5.15±0.5 | 5.24±1 | 0.12 | 0.20 | 0.587 | 0.791 | 0.122 | 0.278 | 0.202 | 0.668 |
| SMARCA4 | 0.1±0.1 | 0.18±0.2 | 0.2±0.2 | 0.08 | 0.11 | 0.138 | 0.441 | 0.129 | 0.284 | 0.95 | 0.982 |
| GAD2 | 0.58±0.3 | 0.62±0.3 | 0.89±0.8 | 0.04 | 0.31 | 0.739 | 0.813 | 0.139 | 0.297 | 0.233 | 0.668 |
| WIF1 | 8.33±1.1 | 8.3±1 | 7.56±1.6 | -0.03 | -0.77 | 0.882 | 0.896 | 0.149 | 0.307 | 0.116 | 0.668 |
| NOTCH2 | 0.16±0.1 | 0.21±0.2 | 0.25±0.2 | 0.05 | 0.09 | 0.342 | 0.673 | 0.182 | 0.364 | 0.713 | 0.98 |
| SOX10 | 0.04±0.1 | 0.03±0 | 0.07±0.1 | -0.01 | 0.03 | 0.526 | 0.767 | 0.194 | 0.377 | 0.451 | 0.902 |
| PMP2 | 5.22±2.8 | 4.74±1.1 | 4.25±2.2 | -0.49 | -0.97 | 0.73 | 0.813 | 0.227 | 0.428 | 0.187 | 0.668 |
| CC2 | 0.03±0.1 | 0.07±0.1 | 0.11±0.2 | 0.03 | 0.07 | 0.29 | 0.661 | 0.245 | 0.45 | 0.891 | 0.982 |
| SLIT2 | 0.03±0.1 | 0.05±0.1 | 0.04±0.1 | 0.01 | 0.00 | 0.415 | 0.74 | 0.331 | 0.565 | 0.924 | 0.982 |
| MGMT | 1.58±1.6 | 1.12±0.7 | 1.14±1 | -0.46 | -0.44 | 0.438 | 0.756 | 0.334 | 0.565 | 0.967 | 0.982 |
| NOTCH4 | 0.05±0.1 | 0.06±0.1 | 0.08±0.1 | 0.02 | 0.03 | 0.535 | 0.767 | 0.356 | 0.565 | 0.595 | 0.935 |
| CNTF | 0.26±0.3 | 0.12±0.1 | 0.15±0.1 | -0.14 | -0.11 | 0.171 | 0.466 | 0.357 | 0.565 | 0.708 | 0.98 |
| IL33 | 0.31±0.2 | 0.4±0.2 | 0.25±0.2 | 0.09 | -0.06 | 0.264 | 0.622 | 0.359 | 0.565 | 0.062 | 0.668 |
| IQGAP1 | 0.07±0.1 | 0.03±0 | 0.07±0.1 | -0.04 | 0.00 | 0.074 | 0.35 | 0.362 | 0.565 | 0.533 | 0.935 |
| BMI1 | 0.07±0.1 | 0.11±0.1 | 0.12±0.2 | 0.05 | 0.06 | 0.181 | 0.466 | 0.37 | 0.565 | 0.799 | 0.982 |
| GLI1 | 0.23±0.2 | 0.25±0.1 | 0.27±0.2 | 0.02 | 0.04 | 0.453 | 0.756 | 0.377 | 0.565 | 0.935 | 0.982 |
| NCAM1 | 9.02±0.1 | 9.01±0.1 | 8.94±0.3 | 0.00 | -0.08 | 0.73 | 0.813 | 0.438 | 0.643 | 0.512 | 0.935 |
| MSI1 | 0.15±0.1 | 0.23±0.4 | 0.21±0.2 | 0.08 | 0.06 | 0.814 | 0.853 | 0.449 | 0.645 | 0.646 | 0.969 |
| EZH2 | 0.12±0.2 | 0.08±0.1 | 0.17±0.2 | -0.04 | 0.04 | 0.498 | 0.767 | 0.462 | 0.649 | 0.177 | 0.668 |
| SMAD9 | 0.2±0.1 | 0.19±0.1 | 0.2±0.2 | -0.02 | -0.01 | 0.751 | 0.813 | 0.515 | 0.709 | 0.868 | 0.982 |
| GMFB | 0.02±0 | 0.05±0.1 | 0.06±0.1 | 0.03 | 0.04 | 0.585 | 0.791 | 0.557 | 0.75 | 0.961 | 0.982 |
| GLI3 | 0.2±0.2 | 0.24±0.2 | 0.13±0.2 | 0.05 | -0.07 | 0.399 | 0.732 | 0.6 | 0.776 | 0.168 | 0.668 |
| PXN | 0.9±1.2 | 0.49±0.5 | 0.58±0.7 | -0.41 | -0.32 | 0.726 | 0.813 | 0.602 | 0.776 | 0.771 | 0.982 |
| POU5F1 | 0.06±0.1 | 0.14±0.2 | 0.07±0.1 | 0.08 | 0.01 | 0.679 | 0.813 | 0.611 | 0.776 | 0.925 | 0.982 |
| FZD9 | 0.06±0.1 | 0.03±0 | 0.05±0.1 | -0.03 | -0.01 | 0.986 | 0.986 | 0.661 | 0.824 | 0.7 | 0.98 |
| CDKN2C | 0.37±0.5 | 0.21±0.1 | 0.23±0.2 | -0.16 | -0.14 | 0.653 | 0.804 | 0.677 | 0.827 | 0.74 | 0.982 |
| SFRP2 | 4.43±0.9 | 4.28±0.9 | 4.23±1.2 | -0.14 | -0.20 | 0.521 | 0.767 | 0.705 | 0.831 | 0.838 | 0.982 |
| RTN4R | 3.5±0.5 | 3.54±0.6 | 3.35±0.7 | 0.04 | -0.15 | 0.856 | 0.883 | 0.705 | 0.831 | 0.512 | 0.935 |
| L1CAM | 3.74±0.4 | 3.75±0.2 | 3.83±0.5 | 0.01 | 0.09 | 0.805 | 0.853 | 0.755 | 0.872 | 0.539 | 0.935 |
| NEUROD1 | 0.03±0 | 0.07±0.1 | 0.02±0.1 | 0.04 | 0.00 | 0.335 | 0.673 | 0.768 | 0.872 | 0.623 | 0.956 |
| OLIG1 | 0.16±0.1 | 0.18±0.1 | 0.18±0.1 | 0.03 | 0.03 | 0.521 | 0.767 | 0.78 | 0.872 | 0.744 | 0.982 |
| FZD4 | 0.52±0.2 | 0.58±0.3 | 0.52±0.2 | 0.06 | 0.00 | 0.347 | 0.673 | 0.882 | 0.971 | 0.367 | 0.793 |
| KLF6 | 0.18±0.2 | 0.27±0.2 | 0.2±0.2 | 0.09 | 0.01 | 0.126 | 0.441 | 0.904 | 0.978 | 0.297 | 0.783 |
| PTCh2 | 0.07±0.1 | 0.08±0.1 | 0.08±0.1 | 0.01 | 0.01 | 0.65 | 0.804 | 0.933 | 0.987 | 0.834 | 0.982 |
| MELK | 0.11±0.1 | 0.07±0.1 | 0.13±0.2 | -0.03 | 0.02 | 0.718 | 0.813 | 0.958 | 0.987 | 0.574 | 0.935 |
| TR4 | 0.01±0 | 0.02±0 | 0.02±0.1 | 0.00 | 0.01 | 0.491 | 0.767 | 0.965 | 0.987 | 0.537 | 0.935 |
| TWIST2 | 0.18±0.2 | 0.32±0.3 | 0.23±0.3 | 0.14 | 0.05 | 0.069 | 0.35 | 0.987 | 0.987 | 0.258 | 0.71 |
| MDGA1 | 5.48±1 | 5.38±0.9 | 5.24±1.5 | -0.10 | -0.24 | 0.61 | 0.804 | 0.987 | 0.987 | 1 | 1 |

D0: preoperatively, POD1; postoperative day 1, POD2: postoperative day 2.

**Supplementary table 3a.** Preoperative levels compared with postoperative levels in patients without spinal cord injury (SCI), serum.

| **Serum protein** | **mean±SD_D0_all** | **mean±SD_D1+D2_neg** | **mean_D1+D2 neg – D0** | **P_D0_all vs. D1+D2 neg** | **FDR_adj_ P_D0_all vs. D1+D2 neg** |
| --- | --- | --- | --- | --- | --- |
| CHI3L1 | 9.97±0.4 | 8.06±0.8 | -1.90 | 2.91E-13 | 1.92E-11 |
| IL6 | 4.04±1.5 | 8.01±1.2 | 3.97 | 1.2E-12 | 3.95E-11 |
| SFRP2 | 4.87±0.6 | 2.88±0.7 | -1.99 | 6.72E-12 | 1.48E-10 |
| RTN4R | 2.5±0.4 | 1.79±0.3 | -0.71 | 1.02E-07 | 1.68E-06 |
| PMP2 | 0.36±0.5 | 1.69±1.1 | 1.33 | 3.04E-06 | 4.02E-05 |
| FABP7 | 0.3±0.3 | 0.05±0.1 | -0.25 | 4.6E-06 | 0.0001 |
| SFRP4 | 2.73±0.4 | 2.3±0.4 | -0.43 | 3.3E-05 | 0.0003 |
| NCAM1 | 9.06±0.2 | 8.86±0.1 | -0.21 | 0.0001 | 0.0005 |
| GPNMB | 6.8±0.4 | 6.41±0.5 | -0.39 | 0.0001 | 0.0008 |
| PTCh2 | 0.34±0.3 | 0.13±0.1 | -0.21 | 0.0002 | 0.0011 |
| MDGA1 | 4.07±0.7 | 3.3±0.6 | -0.77 | 0.0002 | 0.0013 |
| GFAP | 0.24±0.3 | 0.04±0.1 | -0.20 | 0.0003 | 0.0014 |
| MMP2 | 6.37±0.3 | 6.13±0.2 | -0.24 | 0.0004 | 0.0019 |
| VIM | 4.88±1 | 5.73±0.6 | 0.86 | 0.0005 | 0.0022 |
| BMPR1B | 0.42±0.3 | 0.16±0.2 | -0.27 | 0.0005 | 0.0022 |
| FZD9 | 0.38±0.3 | 0.15±0.2 | -0.24 | 0.0006 | 0.0022 |
| ITGA2 | 2.02±0.4 | 2.33±0.3 | 0.31 | 0.0006 | 0.0022 |
| TWIST2 | 0.47±0.6 | 0.15±0.2 | -0.32 | 0.0010 | 0.0038 |
| CSNK2B | 0.37±0.3 | 0.16±0.1 | -0.21 | 0.0016 | 0.0057 |
| BMI1 | 0.47±0.5 | 0.13±0.2 | -0.34 | 0.0019 | 0.0062 |
| OLIG1 | 0.46±0.3 | 0.22±0.2 | -0.24 | 0.0020 | 0.0062 |
| NEUROD1 | 0.33±0.3 | 0.12±0.2 | -0.21 | 0.0021 | 0.0064 |
| SOX2 | 0.4±0.5 | 0.14±0.2 | -0.26 | 0.0027 | 0.0076 |
| TRRAP | 0.5±0.5 | 0.19±0.2 | -0.31 | 0.0028 | 0.0076 |
| GLI2 | 0.36±0.4 | 0.1±0.1 | -0.26 | 0.0030 | 0.0078 |
| SLIT2 | 0.57±0.4 | 0.3±0.3 | -0.27 | 0.0033 | 0.0084 |
| MELK | 0.46±0.5 | 0.16±0.2 | -0.30 | 0.0037 | 0.0089 |
| GMFB | 0.32±0.4 | 0.1±0.2 | -0.22 | 0.0039 | 0.0093 |
| GAD2 | 0.22±0.3 | 0.09±0.2 | -0.13 | 0.0042 | 0.0095 |
| SOX10 | 0.36±0.3 | 0.17±0.2 | -0.18 | 0.0051 | 0.0113 |
| CAMK2G | 0.32±0.3 | 0.12±0.1 | -0.20 | 0.0054 | 0.0115 |
| GLI1 | 0.56±0.5 | 0.3±0.3 | -0.26 | 0.0075 | 0.0155 |
| ITGA6 | 0.83±0.5 | 0.5±0.4 | -0.33 | 0.0127 | 0.0248 |
| FZD2 | 0.65±0.4 | 0.41±0.4 | -0.24 | 0.0128 | 0.0248 |
| FZD4 | 0.84±0.6 | 0.52±0.3 | -0.32 | 0.0204 | 0.0378 |
| CNTF | 0.35±0.3 | 0.2±0.2 | -0.14 | 0.0206 | 0.0378 |
| SIRT2 | 3.73±0.8 | 4.25±0.9 | 0.52 | 0.0244 | 0.0435 |
| SMAD9 | 0.54±0.5 | 0.29±0.3 | -0.26 | 0.0307 | 0.0533 |
| SMARCA4 | 0.28±0.4 | 0.09±0.1 | -0.19 | 0.0330 | 0.0558 |
| PDPN | 0.45±0.4 | 0.24±0.3 | -0.21 | 0.0401 | 0.0661 |

**Supplementary table 3b**. Preoperative levels compared with postoperative levels in patients without SCI, CSF.

| **CSF Protein** | **mean±SD_D0_all** | **mean±SD_**  **D1+D2_neg** | **mean_**  **D1+D2 neg – D0** | **P_D0_all vs. D1+D2 neg** | **FDR_adj_ P_D0_all vs. D1+D2 neg** |
| --- | --- | --- | --- | --- | --- |
| IL6 | 2.69±1.2 | 6.07±1.5 | 3.38 | 2.61E-09 | 1.724E-07 |
| CX3CL1 | 2.96±0.4 | 3.6±0.6 | 0.64 | 0.0001 | 0.0039 |
| CHI3L1 | 9.49±0.2 | 9.07±0.5 | -0.42 | 0.0003 | 0.0072 |
| GFAP | 0.65±0.3 | 1.15±0.6 | 0.50 | 0.0006 | 0.0105 |
| ICAM1 | 9.44±0.7 | 10.06±0.6 | 0.62 | 0.0018 | 0.0216 |
| ITGA2 | 0.27±0.1 | 0.43±0.2 | 0.16 | 0.0020 | 0.0216 |
| VIM | 5.08±1.2 | 4.05±1.1 | -1.03 | 0.0032 | 0.0299 |
| CSPG4 | 0.62±0.3 | 1±0.5 | 0.38 | 0.0037 | 0.0306 |
| DLL1 | 6.35±0.9 | 7.19±0.9 | 0.83 | 0.0043 | 0.0316 |
| GPNMB | 4.71±0.7 | 5.22±0.6 | 0.51 | 0.0117 | 0.0770 |
| FN1 | 4.9±0.6 | 4.46±0.5 | -0.44 | 0.0172 | 0.0947 |
| SFRP4 | 2.6±0.2 | 2.49±0.2 | -0.12 | 0.0172 | 0.0947 |
| FZD2 | 0.27±0.2 | 0.43±0.2 | 0.16 | 0.0231 | 0.1069 |
| CSNK2B | 0.14±0.2 | 0.28±0.3 | 0.14 | 0.0233 | 0.1069 |
| IL1B | 0±0 | 0.2±0.8 | 0.20 | 0.0243 | 0.1069 |
| PDPN | 0.43±0.2 | 0.61±0.3 | 0.19 | 0.0307 | 0.1267 |
| FABP7 | 0.14±0.2 | 0.26±0.3 | 0.12 | 0.0340 | 0.1321 |
| SOX2 | 0.08±0.1 | 0.16±0.2 | 0.08 | 0.0370 | 0.1356 |

**Supplementary table 3c.** Preoperative levels compared with levels in patients with postoperative SCI, serum

| **Serum protein** | **mean±SD_D0_all** | **mean±SD_D1+D2_pos** | **mean_D1+D2 pos – D0** | **P_D0_all vs. D1+D2 pos** | **FDR_adj_ P_D0_all vs. D1+D2 pos** |
| --- | --- | --- | --- | --- | --- |
| CHI3L1 | 9.97±0.4 | 6.37±0.1 | -3.60 | 0.0072 | 0.1196 |
| IL6 | 4.04±1.5 | 10.81±0.4 | 6.77 | 0.0072 | 0.1196 |
| VIM | 4.88±1 | 6.91±0.2 | 2.03 | 0.0072 | 0.1196 |
| MGMT | 4.57±1.2 | 6.73±0.6 | 2.17 | 0.0072 | 0.1196 |
| OLIG1 | 0.46±0.3 | 0.07±0.1 | -0.39 | 0.0145 | 0.1482 |
| SIRT2 | 3.73±0.8 | 6.51±1 | 2.78 | 0.0145 | 0.1482 |
| PMP2 | 0.36±0.5 | 4.52±0.7 | 4.16 | 0.0230 | 0.1482 |
| RTN4R | 2.5±0.4 | 1.65±0.2 | -0.85 | 0.0290 | 0.1482 |
| ITGA2 | 2.02±0.4 | 2.94±0.4 | 0.93 | 0.0290 | 0.1482 |
| SLIT2 | 0.57±0.4 | 0.1±0.1 | -0.47 | 0.0290 | 0.1482 |
| CDCP1 | 4.1±0.8 | 5.14±0.1 | 1.04 | 0.0290 | 0.1482 |
| FABP7 | 0.3±0.3 | 0±0 | -0.30 | 0.0321 | 0.1482 |
| FZD9 | 0.38±0.3 | 0±0 | -0.38 | 0.0321 | 0.1482 |
| TWIST2 | 0.47±0.6 | 0±0 | -0.47 | 0.0321 | 0.1482 |
| GAD2 | 0.22±0.3 | 0.52±0 | 0.30 | 0.0408 | 0.1482 |
| SOX2 | 0.4±0.5 | 0±0 | -0.40 | 0.0412 | 0.1482 |
| IQGAP1 | 0.47±0.4 | 0±0 | -0.47 | 0.0412 | 0.1482 |
| NCAM1 | 9.06±0.2 | 8.81±0.1 | -0.25 | 0.0435 | 0.1482 |
| IL33 | 0.79±0.4 | 1.32±0.3 | 0.52 | 0.0435 | 0.1482 |
| PTCh2 | 0.34±0.3 | 0.04±0.1 | -0.31 | 0.0472 | 0.1482 |
| CNTF | 0.35±0.3 | 0.06±0.1 | -0.29 | 0.0472 | 0.1482 |

**Supplementary table 3d.** Preoperative levels compared with levels in patients with postoperative SCI, CSF.

| **CSF Protein** | **mean±SD_D0_all** | **mean±SD_D1+D2_pos** | **mean_D1+D2 pos – D0** | **P_D0_all vs. D1+D2 pos** | **FDR_adj_ P_D0_all vs. D1+D2 pos** |
| --- | --- | --- | --- | --- | --- |
| IL6 | 2.69±1.2 | 7.97±1.1 | 5.28 | 0.0004 | 0.0245 |
| GFAP | 0.65±0.3 | 3.23±2 | 2.57 | 0.0023 | 0.0734 |
| SOX2 | 0.08±0.1 | 0.27±0.1 | 0.19 | 0.0113 | 0.1697 |
| CSNK2B | 0.14±0.2 | 0.44±0.2 | 0.30 | 0.0129 | 0.1697 |
| VIM | 5.08±1.2 | 3.07±1.1 | -2.01 | 0.0177 | 0.1697 |
| SFRP4 | 2.6±0.2 | 2.12±0.6 | -0.49 | 0.0177 | 0.1697 |
| GMFB | 0.02±0 | 0.09±0.1 | 0.08 | 0.0183 | 0.1697 |

D0: preoperatively, D1+D2: postoperative day 1 and postoperative day 2 combined, neg: no spinal cord injury SCI, pos: spinal cord injury. FDR: false discovery rate.

**Supplementary table 4a.** Preoperative levels compared with postoperative levels in patients without delirium, serum.

| **Serum protein** | **mean±SD_D0_all** | **mean±SD_D1+D2_neg** | **mean_D1+D2 neg – D0** | **P_D0_all vs. D1+D2 neg** | **FDR_adj_ P_D0_all vs. D1+D2 neg** |
| --- | --- | --- | --- | --- | --- |
| CHI3L1 | 9.97±0.4 | 7.95±0.9 | -2.02 | 4.94E-11 | 3.12E-09 |
| IL6 | 4.04±1.5 | 8.17±1.4 | 4.13 | 9.45E-11 | 3.12E-09 |
| SFRP2 | 4.87±0.6 | 2.8±0.6 | -2.07 | 3.07E-10 | 6.76E-09 |
| RTN4R | 2.5±0.4 | 1.78±0.3 | -0.72 | 3.54E-07 | 5.84E-06 |
| FABP7 | 0.3±0.3 | 0.02±0.1 | -0.28 | 6.34E-07 | 8.37E-06 |
| NCAM1 | 9.06±0.2 | 8.85±0.1 | -0.21 | 1.41E-05 | 0.0002 |
| PMP2 | 0.36±0.5 | 1.95±1.5 | 1.59 | 2.15E-05 | 0.0002 |
| PTCh2 | 0.34±0.3 | 0.1±0.1 | -0.25 | 0.0001 | 0.0005 |
| SFRP4 | 2.73±0.4 | 2.32±0.3 | -0.41 | 0.0001 | 0.0005 |
| TWIST2 | 0.47±0.6 | 0.08±0.1 | -0.39 | 0.0001 | 0.0005 |
| SOX2 | 0.4±0.5 | 0.07±0.1 | -0.34 | 0.0001 | 0.0006 |
| FZD9 | 0.38±0.3 | 0.1±0.1 | -0.28 | 0.0001 | 0.0006 |
| BMPR1B | 0.42±0.3 | 0.12±0.2 | -0.31 | 0.0002 | 0.0009 |
| GPNMB | 6.8±0.4 | 6.44±0.4 | -0.36 | 0.0002 | 0.0011 |
| SOX10 | 0.36±0.3 | 0.09±0.2 | -0.27 | 0.0002 | 0.0011 |
| MDGA1 | 4.07±0.7 | 3.28±0.6 | -0.79 | 0.0003 | 0.0013 |
| SLIT2 | 0.57±0.4 | 0.21±0.2 | -0.36 | 0.0004 | 0.0014 |
| NEUROD1 | 0.33±0.3 | 0.08±0.2 | -0.25 | 0.0004 | 0.0014 |
| GMFB | 0.32±0.4 | 0.09±0.2 | -0.23 | 0.0004 | 0.0015 |
| MMP2 | 6.37±0.3 | 6.12±0.2 | -0.25 | 0.0004 | 0.0015 |
| BMI1 | 0.47±0.5 | 0.07±0.1 | -0.40 | 0.0005 | 0.0016 |
| OLIG1 | 0.46±0.3 | 0.19±0.2 | -0.27 | 0.0007 | 0.0020 |
| GLI1 | 0.56±0.5 | 0.25±0.3 | -0.31 | 0.0008 | 0.0022 |
| IQGAP1 | 0.47±0.4 | 0.16±0.2 | -0.31 | 0.0014 | 0.0038 |
| FZD2 | 0.65±0.4 | 0.29±0.2 | -0.35 | 0.0014 | 0.0038 |
| GLI2 | 0.36±0.4 | 0.07±0.1 | -0.29 | 0.0018 | 0.0044 |
| GFAP | 0.24±0.3 | 0.06±0.2 | -0.17 | 0.0020 | 0.0049 |
| CNTF | 0.35±0.3 | 0.15±0.2 | -0.20 | 0.0021 | 0.0049 |
| VIM | 4.88±1 | 5.74±0.7 | 0.86 | 0.0021 | 0.0049 |
| TRRAP | 0.5±0.5 | 0.16±0.1 | -0.34 | 0.0022 | 0.0049 |
| TR4 | 0.7±0.7 | 0.13±0.2 | -0.56 | 0.0025 | 0.0054 |
| MELK | 0.46±0.5 | 0.14±0.2 | -0.32 | 0.0029 | 0.0057 |
| GAD2 | 0.22±0.3 | 0.08±0.2 | -0.14 | 0.0029 | 0.0057 |
| ITGA2 | 2.02±0.4 | 2.32±0.3 | 0.31 | 0.0030 | 0.0057 |
| CAMK2G | 0.32±0.3 | 0.1±0.1 | -0.22 | 0.0032 | 0.0061 |
| FZD4 | 0.84±0.6 | 0.48±0.2 | -0.36 | 0.0050 | 0.0092 |
| SMAD9 | 0.54±0.5 | 0.21±0.2 | -0.34 | 0.0060 | 0.0107 |
| SMARCA4 | 0.28±0.4 | 0.04±0.1 | -0.24 | 0.0071 | 0.0123 |
| ITGA6 | 0.83±0.5 | 0.46±0.4 | -0.37 | 0.0093 | 0.0157 |
| CSNK2B | 0.37±0.3 | 0.19±0.1 | -0.18 | 0.0129 | 0.0212 |
| POU5F1 | 0.31±0.4 | 0.1±0.1 | -0.21 | 0.0219 | 0.0346 |
| EZH2 | 0.66±0.7 | 0.19±0.3 | -0.47 | 0.0220 | 0.0346 |
| PDPN | 0.45±0.4 | 0.21±0.2 | -0.25 | 0.0238 | 0.0366 |
| NOTCH2 | 0.27±0.3 | 0.09±0.1 | -0.18 | 0.0254 | 0.0381 |
| SIRT2 | 3.73±0.8 | 4.3±1 | 0.57 | 0.0329 | 0.0483 |
| DLL1 | 10.61±0.6 | 10.94±0.4 | 0.33 | 0.0484 | 0.0694 |

**Supplementary table 4b.** Preoperative levels compared with postoperative levels in patients without delirium, CSF.

| **CSF protein** | **mean±SD_D0_all** | **mean±SD_**  **D1+D2_neg** | **mean_D1+D2 neg – D0** | **P_D0_all vs. D1+D2 neg** | **FDR_adj_ P_D0_all vs. D1+D2 neg** |
| --- | --- | --- | --- | --- | --- |
| IL6 | 2.69±1.2 | 6.34±1.7 | 3.65 | 7.22E-08 | 4.76E-06 |
| CHI3L1 | 9.49±0.2 | 9.1±0.6 | -0.39 | 0.0085 | 0.0964 |
| CX3CL1 | 2.96±0.4 | 3.39±0.9 | 0.43 | 0.0085 | 0.0964 |
| SOX2 | 0.08±0.1 | 0.19±0.2 | 0.11 | 0.0086 | 0.0964 |
| CSPG4 | 0.62±0.3 | 0.97±0.5 | 0.35 | 0.0134 | 0.0964 |
| GFAP | 0.65±0.3 | 1.32±1.1 | 0.66 | 0.0134 | 0.0964 |
| ITGA2 | 0.27±0.1 | 0.45±0.3 | 0.18 | 0.0134 | 0.0964 |
| CSNK2B | 0.14±0.2 | 0.3±0.2 | 0.17 | 0.0135 | 0.0964 |
| IL1B | 0±0 | 0.28±1 | 0.28 | 0.0138 | 0.0964 |
| SFRP4 | 2.6±0.2 | 2.43±0.3 | -0.18 | 0.0146 | 0.0964 |
| VIM | 5.08±1.2 | 4.04±1.3 | -1.04 | 0.0173 | 0.1040 |
| ICAM1 | 9.44±0.7 | 9.84±1 | 0.40 | 0.0355 | 0.1952 |
| DLL1 | 6.35±0.9 | 6.96±1.4 | 0.61 | 0.0412 | 0.2091 |
| SMARCA4 | 0.1±0.1 | 0.22±0.2 | 0.12 | 0.0470 | 0.2130 |
| FZD2 | 0.27±0.2 | 0.44±0.2 | 0.16 | 0.0484 | 0.2130 |

**Supplementary table 4c.** Preoperative levels compared with postoperative levels in patients with delirium, serum.

| **CSF protein** | **mean±SD_D0_all** | **mean±SD_**  **D1+D2_neg** | **mean_D1+D2 neg – D0** | **P_D0_all vs. D1+D2 neg** | **FDR_adj_ P_D0_all vs. D1+D2 neg** |
| --- | --- | --- | --- | --- | --- |
| IL6 | 2.69±1.2 | 6.34±1.7 | 3.65 | 7.22E-08 | 4.76E-06 |
| CHI3L1 | 9.49±0.2 | 9.1±0.6 | -0.39 | 0.0085 | 0.0964 |
| CX3CL1 | 2.96±0.4 | 3.39±0.9 | 0.43 | 0.0085 | 0.0964 |
| SOX2 | 0.08±0.1 | 0.19±0.2 | 0.11 | 0.0086 | 0.0964 |
| CSPG4 | 0.62±0.3 | 0.97±0.5 | 0.35 | 0.0134 | 0.0964 |
| GFAP | 0.65±0.3 | 1.32±1.1 | 0.66 | 0.0134 | 0.0964 |
| ITGA2 | 0.27±0.1 | 0.45±0.3 | 0.18 | 0.0134 | 0.0964 |
| CSNK2B | 0.14±0.2 | 0.3±0.2 | 0.17 | 0.0135 | 0.0964 |
| IL1B | 0±0 | 0.28±1 | 0.28 | 0.0138 | 0.0964 |
| SFRP4 | 2.6±0.2 | 2.43±0.3 | -0.18 | 0.0146 | 0.0964 |
| VIM | 5.08±1.2 | 4.04±1.3 | -1.04 | 0.0173 | 0.1040 |
| ICAM1 | 9.44±0.7 | 9.84±1 | 0.40 | 0.0355 | 0.1952 |
| DLL1 | 6.35±0.9 | 6.96±1.4 | 0.61 | 0.0412 | 0.2091 |
| SMARCA4 | 0.1±0.1 | 0.22±0.2 | 0.12 | 0.0470 | 0.2130 |
| FZD2 | 0.27±0.2 | 0.44±0.2 | 0.16 | 0.0484 | 0.2130 |

**Supplementary table 4d.** Preoperative levels compared with postoperative levels in patients with delirium, CSF.

| **CSF protein** | **mean±SD_D0_all** | **mean±SD_D1+D2_pos** | **mean_D1+D2 pos – D0** | **P_D0_all vs. D1+D2 pos** | **FDR_adj_ P_D0_all vs. D1+D2 pos** |
| --- | --- | --- | --- | --- | --- |
| IL6 | 2.69±1.2 | 6.3±1.5 | 3.61 | 5.93E-07 | 3.92E-05 |
| GFAP | 0.65±0.3 | 1.59±1.2 | 0.93 | 1.37E-05 | 0.0005 |
| CX3CL1 | 2.96±0.4 | 3.67±0.4 | 0.70 | 0.0002 | 0.0046 |
| CHI3L1 | 9.49±0.2 | 8.99±0.6 | -0.50 | 0.0013 | 0.0166 |
| VIM | 5.08±1.2 | 3.74±0.8 | -1.34 | 0.0013 | 0.0166 |
| ICAM1 | 9.44±0.7 | 10.19±0.6 | 0.75 | 0.0036 | 0.0399 |
| DLL1 | 6.35±0.9 | 7.22±0.7 | 0.87 | 0.0055 | 0.0518 |
| GLI2 | 0.11±0.2 | 0.01±0 | -0.10 | 0.0071 | 0.0587 |
| ITGA2 | 0.27±0.1 | 0.39±0.1 | 0.12 | 0.0105 | 0.0768 |
| FABP7 | 0.14±0.2 | 0.38±0.3 | 0.24 | 0.0147 | 0.0901 |
| GPNMB | 4.71±0.7 | 5.3±0.6 | 0.59 | 0.0150 | 0.0901 |
| CSPG4 | 0.62±0.3 | 1.02±0.5 | 0.40 | 0.0189 | 0.1039 |
| SFRP4 | 2.6±0.2 | 2.45±0.2 | -0.15 | 0.0359 | 0.1758 |
| FZD2 | 0.27±0.2 | 0.43±0.2 | 0.16 | 0.0373 | 0.1758 |

D0: preoperatively, D1+D2: postoperative day 1 and postoperative day 2 combined, neg: no delirium, pos: postoperative delirium. FDR: false discovery rate.

**Supplementary table 5a.** Preoperative levels compared with postoperative levels in patients without hallucinations, serum.

| **Serum_Protein** | **mean±SD_D0_all** | **mean±SD_**  **D1+D2_neg** | **mean_D1+D2 neg – D0** | **P_D0_all vs. D1+D2 neg** | **FDR_adj_ P_D0_all vs. D1+D2 neg** |
| --- | --- | --- | --- | --- | --- |
| CHI3L1 | 9.97±0.4 | 8±0.9 | -1.96 | 7.59063E-10 | 2.33E-08 |
| IL6 | 4.04±1.5 | 7.87±1.1 | 3.83 | 1.0588E-09 | 2.33E-08 |
| SFRP2 | 4.87±0.6 | 2.65±0.8 | -2.22 | 1.0588E-09 | 2.33E-08 |
| RTN4R | 2.5±0.4 | 1.7±0.4 | -0.80 | 6.556E-07 | 1.08E-05 |
| FABP7 | 0.3±0.3 | 0.04±0.1 | -0.26 | 3.51823E-05 | 0.0005 |
| SFRP4 | 2.73±0.4 | 2.2±0.5 | -0.53 | 0.0001 | 0.0010 |
| GPNMB | 6.8±0.4 | 6.29±0.6 | -0.51 | 0.0001 | 0.0010 |
| SLIT2 | 0.57±0.4 | 0.21±0.3 | -0.36 | 0.0001 | 0.0010 |
| MMP2 | 6.37±0.3 | 6.09±0.2 | -0.28 | 0.0002 | 0.0011 |
| BMPR1B | 0.42±0.3 | 0.11±0.1 | -0.32 | 0.0002 | 0.0011 |
| PMP2 | 0.36±0.5 | 1.68±1.3 | 1.32 | 0.0002 | 0.0011 |
| NCAM1 | 9.06±0.2 | 8.85±0.1 | -0.21 | 0.0002 | 0.0011 |
| MDGA1 | 4.07±0.7 | 3.18±0.6 | -0.89 | 0.0004 | 0.0023 |
| PTCh2 | 0.34±0.3 | 0.13±0.1 | -0.22 | 0.0008 | 0.0038 |
| CAMK2G | 0.32±0.3 | 0.07±0.1 | -0.24 | 0.0013 | 0.0059 |
| TWIST2 | 0.47±0.6 | 0.14±0.2 | -0.33 | 0.0015 | 0.0062 |
| GMFB | 0.32±0.4 | 0.08±0.2 | -0.25 | 0.0019 | 0.0071 |
| GLI1 | 0.56±0.5 | 0.21±0.3 | -0.35 | 0.0019 | 0.0071 |
| BMI1 | 0.47±0.5 | 0.11±0.1 | -0.37 | 0.0024 | 0.0082 |
| GLI2 | 0.36±0.4 | 0.08±0.1 | -0.28 | 0.0031 | 0.0101 |
| ITGA2 | 2.02±0.4 | 2.31±0.3 | 0.29 | 0.0032 | 0.0101 |
| ITGA6 | 0.83±0.5 | 0.39±0.4 | -0.45 | 0.0043 | 0.0129 |
| TRRAP | 0.5±0.5 | 0.17±0.1 | -0.33 | 0.0048 | 0.0134 |
| GFAP | 0.24±0.3 | 0.04±0.1 | -0.19 | 0.0049 | 0.0134 |
| FZD2 | 0.65±0.4 | 0.28±0.3 | -0.36 | 0.0055 | 0.0145 |
| GAD2 | 0.22±0.3 | 0.09±0.2 | -0.13 | 0.0073 | 0.0185 |
| SOX2 | 0.4±0.5 | 0.14±0.2 | -0.26 | 0.0079 | 0.0194 |
| FZD9 | 0.38±0.3 | 0.18±0.2 | -0.21 | 0.0095 | 0.0223 |
| CSNK2B | 0.37±0.3 | 0.18±0.2 | -0.19 | 0.0115 | 0.0262 |
| MELK | 0.46±0.5 | 0.17±0.2 | -0.30 | 0.0133 | 0.0292 |
| SMAD9 | 0.54±0.5 | 0.23±0.3 | -0.31 | 0.0147 | 0.0314 |
| OLIG1 | 0.46±0.3 | 0.25±0.2 | -0.21 | 0.0162 | 0.0324 |
| FZD4 | 0.84±0.6 | 0.51±0.3 | -0.33 | 0.0162 | 0.0324 |
| VIM | 4.88±1 | 5.57±0.7 | 0.69 | 0.0174 | 0.0338 |
| NEUROD1 | 0.33±0.3 | 0.15±0.2 | -0.18 | 0.0183 | 0.0346 |
| NOTCH2 | 0.27±0.3 | 0.09±0.1 | -0.18 | 0.0218 | 0.0400 |
| TR4 | 0.7±0.7 | 0.2±0.2 | -0.49 | 0.0229 | 0.0409 |

**Supplementary table 5b.** Preoperative levels compared with postoperative levels in patients without hallucinations, CSF.

| **CSF_Protein** | **mean±SD_D0_all** | **mean±SD_**  **D1+D2_neg** | **mean_D1+D2 neg – D0** | **P_D0_all vs. D1+D2 neg** | **FDR_adj_ P_D0_all vs. D1+D2 neg** |
| --- | --- | --- | --- | --- | --- |
| IL6 | 2.69±1.2 | 6.71±1.2 | 4.02 | 3.98595E-07 | 2.63E-05 |
| ITGA2 | 0.27±0.1 | 0.51±0.2 | 0.24 | 0.0002 | 0.0070 |
| CX3CL1 | 2.96±0.4 | 3.7±0.5 | 0.74 | 0.0004 | 0.0082 |
| DLL1 | 6.35±0.9 | 7.52±1 | 1.17 | 0.0013 | 0.0207 |
| IL1B | 0±0 | 0.43±1.2 | 0.43 | 0.0023 | 0.0258 |
| CSPG4 | 0.62±0.3 | 1.12±0.5 | 0.49 | 0.0023 | 0.0258 |
| GFAP | 0.65±0.3 | 1.67±1.6 | 1.02 | 0.0031 | 0.0297 |
| SOX2 | 0.08±0.1 | 0.2±0.1 | 0.12 | 0.0042 | 0.0343 |
| ICAM1 | 9.44±0.7 | 10.2±0.7 | 0.76 | 0.0048 | 0.0352 |
| CC2 | 0.03±0.1 | 0.15±0.2 | 0.12 | 0.0065 | 0.0394 |
| BMPR1B | 0.29±0.2 | 0.51±0.2 | 0.22 | 0.0068 | 0.0394 |
| CDCP1 | 3.63±0.7 | 4.36±0.7 | 0.73 | 0.0072 | 0.0394 |
| GPNMB | 4.71±0.7 | 5.39±0.6 | 0.68 | 0.0081 | 0.0414 |
| CHI3L1 | 9.49±0.2 | 8.97±0.7 | -0.53 | 0.0105 | 0.0494 |
| BMI1 | 0.07±0.1 | 0.17±0.1 | 0.10 | 0.0132 | 0.0579 |
| SMARCA4 | 0.1±0.1 | 0.23±0.1 | 0.13 | 0.0145 | 0.0597 |
| FN1 | 4.9±0.6 | 4.36±0.6 | -0.54 | 0.0169 | 0.0654 |
| ITGA6 | 0.08±0.2 | 0.18±0.2 | 0.10 | 0.0221 | 0.0811 |
| TWIST2 | 0.18±0.2 | 0.37±0.3 | 0.19 | 0.0249 | 0.0866 |
| CD44 | 6.26±0.2 | 6.41±0.1 | 0.15 | 0.0263 | 0.0866 |
| CSNK2B | 0.14±0.2 | 0.3±0.2 | 0.17 | 0.0312 | 0.0931 |
| FABP7 | 0.14±0.2 | 0.31±0.3 | 0.17 | 0.0330 | 0.0931 |
| FZD2 | 0.27±0.2 | 0.45±0.1 | 0.17 | 0.0339 | 0.0931 |
| PDPN | 0.43±0.2 | 0.71±0.4 | 0.28 | 0.0339 | 0.0931 |
| GAD2 | 0.58±0.3 | 1.02±0.8 | 0.44 | 0.0438 | 0.1111 |
| NF1 | 5±0.1 | 4.87±0.2 | -0.13 | 0.0438 | 0.1111 |
| NOTCH4 | 0.05±0.1 | 0.12±0.1 | 0.07 | 0.0458 | 0.1120 |
| TRRAP | 0.07±0.1 | 0.16±0.2 | 0.09 | 0.0478 | 0.1126 |

**Supplementary table 5c.** Preoperative levels compared with levels in patients with postoperative hallucinations, serum.

| **Serum_**  **Protein** | **mean±SD_**  **D0_all** | **mean±SD_**  **D1+D2_pos** | **mean_D1+D2 pos – D0** | **P_D0_all vs. D1+D2 pos** | **FDR_adj_ P_D0_all vs. D1+D2 pos** |
| --- | --- | --- | --- | --- | --- |
| CHI3L1 | 9.97±0.4 | 7.93±0.8 | -2.03 | 2.74396E-10 | 1.81E-08 |
| IL6 | 4.04±1.5 | 8.51±1.5 | 4.47 | 1.76398E-09 | 5.82E-08 |
| SFRP2 | 4.87±0.6 | 3.26±0.6 | -1.61 | 6.25623E-08 | 1.38E-06 |
| PMP2 | 0.36±0.5 | 2.04±1.3 | 1.68 | 6.73732E-06 | 0.000111 |
| RTN4R | 2.5±0.4 | 1.88±0.3 | -0.62 | 2.90239E-05 | 0.000383 |
| VIM | 4.88±1 | 6.06±0.6 | 1.18 | 0.0001 | 0.0006 |
| FABP7 | 0.3±0.3 | 0.06±0.1 | -0.24 | 0.0002 | 0.0022 |
| FZD9 | 0.38±0.3 | 0.1±0.1 | -0.29 | 0.0003 | 0.0024 |
| NCAM1 | 9.06±0.2 | 8.86±0.1 | -0.20 | 0.0010 | 0.0070 |
| PTCh2 | 0.34±0.3 | 0.13±0.1 | -0.22 | 0.0011 | 0.0070 |
| SOX10 | 0.36±0.3 | 0.11±0.2 | -0.24 | 0.0013 | 0.0070 |
| OLIG1 | 0.46±0.3 | 0.17±0.2 | -0.29 | 0.0013 | 0.0070 |
| ITGA2 | 2.02±0.4 | 2.43±0.4 | 0.41 | 0.0014 | 0.0070 |
| NEUROD1 | 0.33±0.3 | 0.07±0.1 | -0.26 | 0.0018 | 0.0086 |
| CNTF | 0.35±0.3 | 0.13±0.1 | -0.22 | 0.0022 | 0.0096 |
| SFRP4 | 2.73±0.4 | 2.45±0.3 | -0.28 | 0.0024 | 0.0098 |
| SIRT2 | 3.73±0.8 | 4.58±0.9 | 0.84 | 0.0026 | 0.0103 |
| MDGA1 | 4.07±0.7 | 3.45±0.5 | -0.62 | 0.0032 | 0.0119 |
| CSNK2B | 0.37±0.3 | 0.16±0.1 | -0.21 | 0.0050 | 0.0173 |
| MELK | 0.46±0.5 | 0.14±0.2 | -0.32 | 0.0057 | 0.0188 |
| SOX2 | 0.4±0.5 | 0.12±0.2 | -0.28 | 0.0064 | 0.0200 |
| GPNMB | 6.8±0.4 | 6.58±0.2 | -0.22 | 0.0070 | 0.0211 |
| TWIST2 | 0.47±0.6 | 0.16±0.2 | -0.31 | 0.0077 | 0.0221 |
| NF1 | 4.8±1.6 | 6.01±1.3 | 1.21 | 0.0101 | 0.0277 |
| TRRAP | 0.5±0.5 | 0.19±0.2 | -0.31 | 0.0133 | 0.0352 |
| GFAP | 0.24±0.3 | 0.11±0.2 | -0.13 | 0.0167 | 0.0423 |
| MGMT | 4.57±1.2 | 5.44±0.8 | 0.87 | 0.0214 | 0.0522 |
| BMI1 | 0.47±0.5 | 0.16±0.2 | -0.31 | 0.0250 | 0.0588 |
| BMPR1B | 0.42±0.3 | 0.21±0.2 | -0.21 | 0.0281 | 0.0640 |
| PXN | 3.15±0.9 | 3.59±0.6 | 0.44 | 0.0291 | 0.0640 |
| GLI2 | 0.36±0.4 | 0.12±0.1 | -0.23 | 0.0435 | 0.0925 |

**Supplementary table 5d.** Preoperative levels compared with levels in patients with postoperative hallucinations, CSF.

| **CSF_Protein** | **mean±SD_**  **D0_all** | **mean±SD_**  **D1+D2_pos** | **mean_D1+D2 pos – D0** | **P_D0_all vs. D1+D2 pos** | **FDR_adj_ P_D0_all vs. D1+D2 pos** |
| --- | --- | --- | --- | --- | --- |
| IL6 | 2.69±1.2 | 6.07±1.7 | 3.38 | 9.51218E-08 | 6.28E-06 |
| VIM | 5.08±1.2 | 3.61±1 | -1.47 | 0.0002 | 0.0074 |
| GFAP | 0.65±0.3 | 1.26±0.7 | 0.61 | 0.0007 | 0.0160 |
| CHI3L1 | 9.49±0.2 | 9.12±0.5 | -0.37 | 0.0019 | 0.0306 |
| CX3CL1 | 2.96±0.4 | 3.37±0.8 | 0.40 | 0.0064 | 0.0848 |
| SFRP4 | 2.6±0.2 | 2.41±0.3 | -0.20 | 0.0078 | 0.0855 |
| CSNK2B | 0.14±0.2 | 0.3±0.3 | 0.17 | 0.0206 | 0.1939 |
| GLI2 | 0.11±0.2 | 0.03±0.1 | -0.08 | 0.0304 | 0.2235 |
| ICAM1 | 9.44±0.7 | 9.83±1 | 0.39 | 0.0305 | 0.2235 |
| IQGAP1 | 0.07±0.1 | 0.04±0.1 | -0.03 | 0.0438 | 0.2850 |
| CSPG4 | 0.62±0.3 | 0.91±0.5 | 0.28 | 0.0476 | 0.2850 |

D0: preoperatively, D1+D2: postoperative day 1 and postoperative day 2 combined, neg: no hallucinations, pos: postoperative hallucinations. FDR: false discovery rate.

**Supplementary table 6a.** Postoperative levels in patients with and without postoperative headache, serum.

| **Serum protein** | **mean±SD_**  **D1+D2_neg** | **mean±SD_**  **D1+D2_pos** | **mean_**  **D1+D2 pos – D1+D2 neg** | **P_D1+D2 neg vs. D1+D2 pos** | **FDR_adj_ P_D1+D2 neg vs. D1+D2 pos** |
| --- | --- | --- | --- | --- | --- |
| SFRP2 | 3.11±0.8 | 2.3±0.4 | -0.81 | 0.0007 | 0.0436 |
| SMAD9 | 0.37±0.3 | 0.07±0.1 | -0.30 | 0.0043 | 0.1113 |
| TR4 | 0.49±0.6 | 0.07±0.1 | -0.42 | 0.0079 | 0.1113 |
| ITGA2 | 2.44±0.3 | 2.1±0.3 | -0.33 | 0.0094 | 0.1113 |
| CSPG4 | 0.71±0.3 | 0.47±0.2 | -0.24 | 0.0107 | 0.1113 |
| SIRT2 | 4.58±1.1 | 3.62±0.6 | -0.96 | 0.0107 | 0.1113 |
| GLI1 | 0.35±0.3 | 0.08±0.1 | -0.27 | 0.0135 | 0.1113 |
| NF1 | 5.36±1.5 | 3.92±0.8 | -1.43 | 0.0135 | 0.1113 |
| NOTCH2 | 0.16±0.2 | 0.02±0 | -0.14 | 0.0180 | 0.1195 |
| BMPR1B | 0.18±0.2 | 0.04±0 | -0.14 | 0.0195 | 0.1195 |
| BMI1 | 0.16±0.2 | 0.02±0 | -0.13 | 0.0199 | 0.1195 |
| VIM | 5.92±0.7 | 5.36±0.6 | -0.56 | 0.0289 | 0.1587 |
| CSNK2B | 0.19±0.1 | 0.08±0.1 | -0.12 | 0.0379 | 0.1811 |
| DLL1 | 10.99±0.4 | 10.69±0.5 | -0.29 | 0.0388 | 0.1811 |
| MGMT | 5.01±1.3 | 4.11±0.9 | -0.90 | 0.0427 | 0.1811 |
| FZD2 | 0.46±0.4 | 0.18±0.2 | -0.28 | 0.0439 | 0.1811 |

**Supplementary table 6b.** Postoperative levels in patients with and without postoperative headache, CSF.

| **CSF protein** | **mean±SD_**  **D1+D2_neg** | **mean±SD_**  **D1+D2_pos** | **mean_D1+D2 pos – D1+D2 neg** | **P_D1+D2 neg vs. D1+D2 pos** | **FDR_adj_ P_D1+D2 neg vs. D1+D2 pos** |
| --- | --- | --- | --- | --- | --- |
| RTN4R | 3.29±0.7 | 4.06±0.1 | 0.77 | 0.0001 | 0.0067 |
| IL1B | 0.01±0 | 0.83±1.7 | 0.83 | 0.0010 | 0.0221 |
| WIF1 | 7.61±1.3 | 9.24±0.6 | 1.63 | 0.0010 | 0.0221 |
| MDGA1 | 5.07±1.2 | 6.26±0.5 | 1.19 | 0.0108 | 0.1790 |
| CDCP1 | 3.81±0.9 | 4.69±0.5 | 0.88 | 0.0211 | 0.2323 |
| DLL1 | 6.82±1.1 | 8.04±0.8 | 1.22 | 0.0246 | 0.2323 |
| SFRP4 | 2.4±0.3 | 2.61±0.1 | 0.21 | 0.0246 | 0.2323 |
| FN1 | 4.63±0.6 | 4.03±0.4 | -0.59 | 0.0286 | 0.2363 |
| GPNMB | 4.97±0.9 | 5.7±0.4 | 0.73 | 0.0331 | 0.2429 |

**Supplementary table 6c.** Preoperative levels compared with postoperative levels in patients without headache, serum.

| **Serum protein** | **mean±SD_**  **D0_all** | **mean±SD_**  **D1+D2_neg** | **mean_D1+D2 neg – D0** | **P_D0_all vs. D1+D2 neg** | **FDR_adj_ P_D0_all vs. D1+D2 neg** |
| --- | --- | --- | --- | --- | --- |
| CHI3L1 | 9.97±0.4 | 7.88±0.8 | -2.09 | 3.84E-13 | 2.54E-11 |
| IL6 | 4.04±1.5 | 8.15±1.4 | 4.11 | 1.17E-11 | 3.87E-10 |
| SFRP2 | 4.87±0.6 | 3.11±0.8 | -1.76 | 2.36E-10 | 5.19E-09 |
| PMP2 | 0.36±0.5 | 1.9±1.2 | 1.54 | 4.89E-07 | 8.07E-06 |
| RTN4R | 2.5±0.4 | 1.84±0.3 | -0.67 | 7.89E-07 | 1.04E-05 |
| FABP7 | 0.3±0.3 | 0.05±0.1 | -0.24 | 1.37E-05 | 0.0002 |
| ITGA2 | 2.02±0.4 | 2.44±0.3 | 0.42 | 6.39E-05 | 0.0006 |
| VIM | 4.88±1 | 5.92±0.7 | 1.04 | 7.70E-05 | 0.0006 |
| NCAM1 | 9.06±0.2 | 8.86±0.1 | -0.20 | 0.0002 | 0.0014 |
| PTCh2 | 0.34±0.3 | 0.13±0.1 | -0.21 | 0.0002 | 0.0016 |
| GPNMB | 6.8±0.4 | 6.48±0.5 | -0.32 | 0.0004 | 0.0020 |
| MDGA1 | 4.07±0.7 | 3.38±0.5 | -0.69 | 0.0004 | 0.0020 |
| SFRP4 | 2.73±0.4 | 2.36±0.4 | -0.37 | 0.0004 | 0.0020 |
| FZD9 | 0.38±0.3 | 0.14±0.1 | -0.25 | 0.0005 | 0.0021 |
| OLIG1 | 0.46±0.3 | 0.2±0.2 | -0.26 | 0.0009 | 0.0040 |
| NEUROD1 | 0.33±0.3 | 0.09±0.2 | -0.23 | 0.0011 | 0.0044 |
| SIRT2 | 3.73±0.8 | 4.58±1.1 | 0.85 | 0.0012 | 0.0046 |
| MELK | 0.46±0.5 | 0.14±0.2 | -0.32 | 0.0021 | 0.0077 |
| GFAP | 0.24±0.3 | 0.09±0.2 | -0.15 | 0.0025 | 0.0085 |
| BMPR1B | 0.42±0.3 | 0.18±0.2 | -0.24 | 0.0037 | 0.0122 |
| SOX10 | 0.36±0.3 | 0.16±0.2 | -0.19 | 0.0043 | 0.0134 |
| TWIST2 | 0.47±0.6 | 0.18±0.3 | -0.29 | 0.0045 | 0.0134 |
| TRRAP | 0.5±0.5 | 0.19±0.2 | -0.31 | 0.0053 | 0.0153 |
| CSNK2B | 0.37±0.3 | 0.19±0.1 | -0.18 | 0.0076 | 0.0210 |
| MMP2 | 6.37±0.3 | 6.17±0.2 | -0.20 | 0.0086 | 0.0227 |
| CNTF | 0.35±0.3 | 0.18±0.2 | -0.16 | 0.0099 | 0.0248 |
| GLI2 | 0.36±0.4 | 0.11±0.1 | -0.25 | 0.0108 | 0.0248 |
| BMI1 | 0.47±0.5 | 0.16±0.2 | -0.31 | 0.0109 | 0.0248 |
| SOX2 | 0.4±0.5 | 0.16±0.2 | -0.24 | 0.0109 | 0.0248 |
| SLIT2 | 0.57±0.4 | 0.32±0.3 | -0.24 | 0.0134 | 0.0295 |
| DLL1 | 10.61±0.6 | 10.99±0.4 | 0.38 | 0.0160 | 0.0342 |
| CAMK2G | 0.32±0.3 | 0.14±0.2 | -0.18 | 0.0211 | 0.0435 |
| PXN | 3.15±0.9 | 3.61±0.8 | 0.46 | 0.0233 | 0.0466 |
| FZD2 | 0.65±0.4 | 0.46±0.4 | -0.19 | 0.0407 | 0.0785 |
| GLI1 | 0.56±0.5 | 0.35±0.3 | -0.21 | 0.0426 | 0.0785 |
| GAD2 | 0.22±0.3 | 0.13±0.2 | -0.09 | 0.0428 | 0.0785 |

**Supplementary table 6d.** Preoperative levels compared with postoperative levels in patients without headache, CSF.

| **CSF protein** | **mean±SD_**  **D0_all** | **mean±SD_**  **D1+D2_neg** | **mean_D1+D2 neg – D0** | **P_D0_all vs. D1+D2 neg** | **FDR_adj_ P_D0_all vs. D1+D2 neg** |
| --- | --- | --- | --- | --- | --- |
| IL6 | 2.69±1.2 | 6.22±1.7 | 3.52 | 5.12E-09 | 3.38E-07 |
| GFAP | 0.65±0.3 | 1.56±1.2 | 0.91 | 0.0001 | 0.0048 |
| VIM | 5.08±1.2 | 3.7±1 | -1.38 | 0.0002 | 0.0049 |
| CHI3L1 | 9.49±0.2 | 9.09±0.6 | -0.40 | 0.0021 | 0.0251 |
| SFRP4 | 2.6±0.2 | 2.4±0.3 | -0.21 | 0.0021 | 0.0251 |
| CX3CL1 | 2.96±0.4 | 3.39±0.8 | 0.43 | 0.0023 | 0.0251 |
| ITGA2 | 0.27±0.1 | 0.42±0.2 | 0.15 | 0.0118 | 0.0900 |
| GLI2 | 0.11±0.2 | 0.02±0.1 | -0.08 | 0.0129 | 0.0900 |
| CSNK2B | 0.14±0.2 | 0.3±0.3 | 0.17 | 0.0130 | 0.0900 |
| ICAM1 | 9.44±0.7 | 9.89±0.9 | 0.45 | 0.0136 | 0.0900 |
| CSPG4 | 0.62±0.3 | 0.92±0.5 | 0.30 | 0.0168 | 0.0981 |
| SOX2 | 0.08±0.1 | 0.18±0.2 | 0.10 | 0.0178 | 0.0981 |
| FZD2 | 0.27±0.2 | 0.42±0.2 | 0.15 | 0.0329 | 0.1670 |
| FABP7 | 0.14±0.2 | 0.27±0.3 | 0.13 | 0.0364 | 0.1717 |
| DLL1 | 6.35±0.9 | 6.82±1.1 | 0.47 | 0.0406 | 0.1787 |
| PDPN | 0.43±0.2 | 0.58±0.3 | 0.16 | 0.0490 | 0.2022 |

**Supplementary table 6e.** Preoperative levels compared with postoperative levels in patients with headache, serum.

| **Serum protein** | **mean±SD_**  **D0_all** | **mean±SD_**  **D1+D2_pos** | **mean_D1+D2 pos – D0** | **P_D0_all vs. D1+D2 pos** | **FDR_adj_ P_D0_all vs. D1+D2 pos** |
| --- | --- | --- | --- | --- | --- |
| SFRP2 | 4.87±0.6 | 2.3±0.4 | -2.57 | 1.37E-06 | 7.89E-05 |
| IL6 | 4.04±1.5 | 8.22±1.1 | 4.18 | 2.39E-06 | 7.89E-05 |
| CHI3L1 | 9.97±0.4 | 8.3±0.9 | -1.67 | 2.29E-05 | 0.0005 |
| RTN4R | 2.5±0.4 | 1.6±0.3 | -0.90 | 4.65E-05 | 0.0008 |
| SFRP4 | 2.73±0.4 | 2.14±0.4 | -0.59 | 0.0003 | 0.0036 |
| BMPR1B | 0.42±0.3 | 0.04±0 | -0.38 | 0.0007 | 0.0068 |
| GLI1 | 0.56±0.5 | 0.08±0.1 | -0.48 | 0.0008 | 0.0068 |
| TWIST2 | 0.47±0.6 | 0.03±0.1 | -0.44 | 0.0009 | 0.0068 |
| SLIT2 | 0.57±0.4 | 0.15±0.1 | -0.42 | 0.0013 | 0.0068 |
| NCAM1 | 9.06±0.2 | 8.82±0.1 | -0.24 | 0.0014 | 0.0068 |
| MMP2 | 6.37±0.3 | 6.08±0.1 | -0.29 | 0.0014 | 0.0068 |
| FABP7 | 0.3±0.3 | 0.03±0.1 | -0.26 | 0.0014 | 0.0068 |
| SOX2 | 0.4±0.5 | 0.02±0 | -0.38 | 0.0014 | 0.0068 |
| SMAD9 | 0.54±0.5 | 0.07±0.1 | -0.47 | 0.0014 | 0.0068 |
| BMI1 | 0.47±0.5 | 0.02±0 | -0.45 | 0.0021 | 0.0089 |
| NOTCH2 | 0.27±0.3 | 0.02±0 | -0.25 | 0.0022 | 0.0089 |
| GPNMB | 6.8±0.4 | 6.22±0.6 | -0.58 | 0.0036 | 0.0136 |
| GMFB | 0.32±0.4 | 0.02±0 | -0.30 | 0.0037 | 0.0136 |
| TR4 | 0.7±0.7 | 0.07±0.1 | -0.62 | 0.0052 | 0.0181 |
| CSNK2B | 0.37±0.3 | 0.08±0.1 | -0.29 | 0.0055 | 0.0181 |
| MDGA1 | 4.07±0.7 | 3.03±0.7 | -1.04 | 0.0072 | 0.0215 |
| ITGA6 | 0.83±0.5 | 0.27±0.3 | -0.56 | 0.0073 | 0.0215 |
| FZD2 | 0.65±0.4 | 0.18±0.2 | -0.47 | 0.0075 | 0.0215 |
| CAMK2G | 0.32±0.3 | 0.06±0.1 | -0.26 | 0.0083 | 0.0229 |
| PTCh2 | 0.34±0.3 | 0.1±0.2 | -0.24 | 0.0091 | 0.0240 |
| FZD4 | 0.84±0.6 | 0.43±0.2 | -0.41 | 0.0099 | 0.0252 |
| GLI2 | 0.36±0.4 | 0.07±0.1 | -0.29 | 0.0116 | 0.0283 |
| TRRAP | 0.5±0.5 | 0.13±0.2 | -0.37 | 0.0132 | 0.0306 |
| PDPN | 0.45±0.4 | 0.12±0.2 | -0.33 | 0.0134 | 0.0306 |
| FZD9 | 0.38±0.3 | 0.15±0.2 | -0.24 | 0.0291 | 0.0641 |
| CSPG4 | 0.73±0.3 | 0.47±0.2 | -0.25 | 0.0308 | 0.0655 |
| SMARCA4 | 0.28±0.4 | 0.03±0.1 | -0.25 | 0.0372 | 0.0766 |
| IQGAP1 | 0.47±0.4 | 0.21±0.4 | -0.26 | 0.0409 | 0.0818 |
| GAD2 | 0.22±0.3 | 0.06±0.1 | -0.16 | 0.0448 | 0.0869 |
| PMP2 | 0.36±0.5 | 1.64±1.6 | 1.29 | 0.0485 | 0.0915 |

**Table 6f.** Preoperative levels compared with postoperative levels in patients with headache, CSF.

| **CSF protein** | **mean±SD_**  **D0_all** | **mean±SD_**  **D1+D2_pos** | **mean_D1+D2 pos – D0** | **P_D0_all vs. D1+D2 pos** | **FDR_adj_ P_D0_all vs. D1+D2 pos** |
| --- | --- | --- | --- | --- | --- |
| IL6 | 2.69±1.2 | 6.77±0.7 | 4.07 | 6.08E-05 | 0.0040 |
| IL1B | 0±0 | 0.83±1.7 | 0.83 | 0.0001 | 0.0043 |
| DLL1 | 6.35±0.9 | 8.04±0.8 | 1.69 | 0.0004 | 0.0084 |
| CX3CL1 | 2.96±0.4 | 3.95±0.5 | 0.98 | 0.0006 | 0.0092 |
| CDCP1 | 3.63±0.7 | 4.69±0.5 | 1.06 | 0.0015 | 0.0194 |
| FN1 | 4.9±0.6 | 4.03±0.4 | -0.87 | 0.0020 | 0.0217 |
| GPNMB | 4.71±0.7 | 5.7±0.4 | 0.99 | 0.0034 | 0.0318 |
| ITGA2 | 0.27±0.1 | 0.47±0.2 | 0.20 | 0.0069 | 0.0457 |
| CSPG4 | 0.62±0.3 | 1.25±0.5 | 0.63 | 0.0069 | 0.0457 |
| CD44 | 6.26±0.2 | 6.45±0.1 | 0.19 | 0.0069 | 0.0457 |
| CHI3L1 | 9.49±0.2 | 8.93±0.5 | -0.56 | 0.0107 | 0.0588 |
| ICAM1 | 9.44±0.7 | 10.33±0.6 | 0.89 | 0.0107 | 0.0588 |
| IQGAP1 | 0.07±0.1 | 0±0 | -0.07 | 0.0123 | 0.0623 |
| NF1 | 5±0.1 | 4.81±0.2 | -0.19 | 0.0194 | 0.0914 |
| RTN4R | 3.5±0.5 | 4.06±0.1 | 0.56 | 0.0279 | 0.1149 |
| SIRT2 | 3.22±1.5 | 3.87±0.7 | 0.65 | 0.0279 | 0.1149 |
| MMP2 | 5.04±0.6 | 5.63±0.4 | 0.59 | 0.0331 | 0.1284 |
| CC2 | 0.03±0.1 | 0.15±0.1 | 0.12 | 0.0477 | 0.1750 |

D0: preoperatively, D1+D2: postoperative day 1 and postoperative day 2 combined, neg: no headache, pos: post-operative headache. FDR: false discovery rate.
